# Synapses learn to utilize stochastic pre-synaptic release for the prediction of postsynaptic dynamics

**DOI:** 10.1371/journal.pcbi.1012531

**Published:** 2024-11-04

**Authors:** David Kappel, Christian Tetzlaff

**Affiliations:** 1 III. Physikalisches Institut – Biophysik, Georg-August Universität, Göttingen, Germany; 2 Institut für Neuroinformatik, Ruhr-Universität Bochum, Bochum, Germany; 3 Group of Computational Synaptic Physiology, Department for Neuro- and Sensory Physiology, University Medical Center Göttingen, Göttingen, Germany; Ernst-Strungmann-Institut, GERMANY

## Abstract

Synapses in the brain are highly noisy, which leads to a large trial-by-trial variability. Given how costly synapses are in terms of energy consumption these high levels of noise are surprising. Here we propose that synapses use noise to represent uncertainties about the somatic activity of the postsynaptic neuron. To show this, we developed a mathematical framework, in which the synapse as a whole interacts with the soma of the postsynaptic neuron in a similar way to an agent that is situated and behaves in an uncertain, dynamic environment. This framework suggests that synapses use an implicit internal model of the somatic membrane dynamics that is being updated by a synaptic learning rule, which resembles experimentally well-established LTP/LTD mechanisms. In addition, this approach entails that a synapse utilizes its inherently noisy synaptic release to also encode its uncertainty about the state of the somatic potential. Although each synapse strives for predicting the somatic dynamics of its postsynaptic neuron, we show that the emergent dynamics of many synapses in a neuronal network resolve different learning problems such as pattern classification or closed-loop control in a dynamic environment. Hereby, synapses coordinate themselves to represent and utilize uncertainties on the network level in behaviorally ambiguous situations.

## Author summary

Synapses in the brain are highly noisy leading to a large trial-by-trial variability in their response to presynaptic action potentials. Adopting the principle of predictive processing, which is based on the idea that agents instantiate an internal model of their environment enabling them to make predictions, take actions, and to minimize surprise, we can show that a synapse utilizes its inherently noisy release to encode its uncertainty about the state of its cellular, postsynaptic environment. The resulting model framework leads to a synaptic learning rule that adapts the synaptic efficacy such that the synapse can predict future postsynaptic spiking. This synaptic learning rule resembles experimentally well-established plasticity mechanisms and makes very precise experimental predictions. In addition, we show that this synaptic learning rule enables neuronal networks to utilize the unreliability of synaptic release to master behaviorally ambiguous situations.

## Introduction

Synapses are inherently unreliable in transmitting their input to the post-synaptic neuron. For example, the probability of neurotransmitter release is typically around 50% [1–3] and can be as low as 20% *in vivo* [4]. In other words, up to 80% of synaptic transmissions fail due to release unreliability, providing one of the major sources of noise in the synapse. Pre- and post-synaptic noise sources result in a large trial-by-trial variability in the post-synaptic current (PSC) [5]. At the same time, synapses are very demanding in terms of energy consumption [6], suggesting that a large portion of the body’s energy intake dissipates by the unreliability of synaptic transmission. Similar to biological synapses also neuromorphic technologies are exposed to noise culminating in unreliable synaptic transmission [7–9]. The functional implication of noisy synaptic transmission, and whether it is a feature or bug in biological and artificial neuronal systems, is therefore highly debated [5, 10–13]. Here, we show that synapses can exploit noisy synaptic transmission to encode their uncertainty about the somatic membrane potential of the postsynaptic neuron. With each synapse doing this, we further show that this enables a neuronal network to encode and utilize uncertainties.

**Fig 1:**
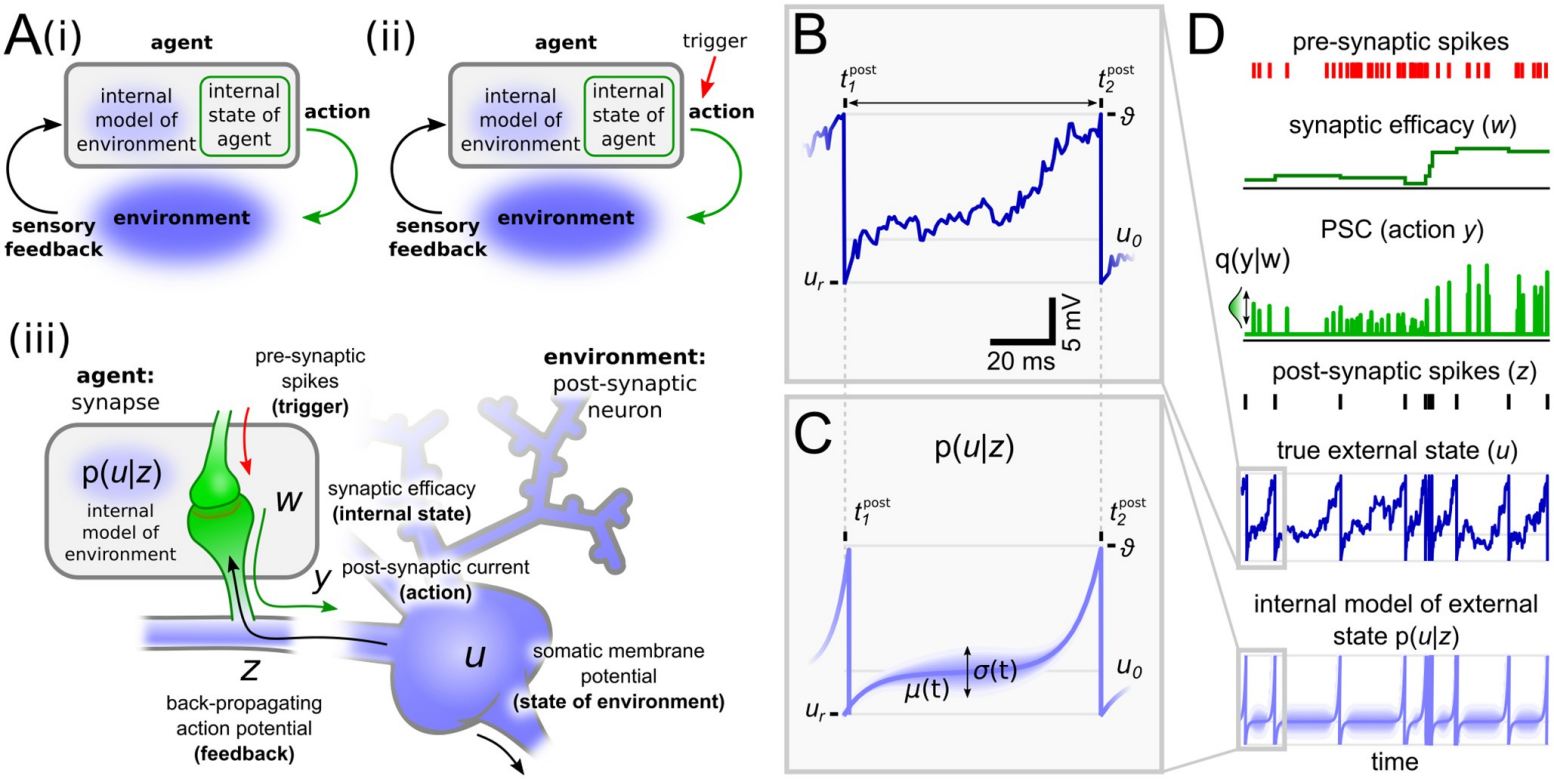
Predictive processing for individual synapses. **A:** i) An agent interacting with the environment through actions, which are determined by the agent’s internal state. Sensory feedback from the environment to the agent is used to update the agent’s internal model of the environment. ii) An additional, external trigger can be included into the framework from i) that determines when actions are initialized. iii) The framework shown in ii) can be transferred to a synapse that interacts with its postsynaptic soma. Relevant variables are the synaptic efficacy (internal state), the postsynaptic current (action), the somatic membrane potential (environmental state) of the postsynaptic neuron (environment), and the back-propagating action potential (feedback). **B:** A single trajectory of the somatic membrane potential *u*(*t*) between two action potentials. **C:** The internal synaptic model of the somatic membrane potential can be characterized by the stochastic bridge model, providing the probability distribution *p*(*u* | *z*) about the value of *u*(*t*) between two postsynaptic spikes. The solid blue line shows the mean, std indicated by shaded area. **D:** Illustration of relevant dynamics. Pre-synaptic input spikes (red) trigger synapses to release stochastic postsynaptic currents *y* (light green) with a mean and variance of pulse scales dependent on the synaptic efficacy *w*. Theoretical Dirac delta pulses are illustrated here as scaled unit-sized pulses. Postsynaptic spike timings reach the synapse through bAPs *z*, constraining the internal model of the somatic membrane potential to the firing threshold *ϑ* and then reset to *u*_*r*_ immediately after every bAP (see panel C). Between bAPs, the internal model estimates the probability density of the membrane potential according to the stochastic process (*μ*(*t*), *σ*(*t*)).

To establish this result, we rely on the predictive processing model framework to describe biological systems that act in uncertain environments. Predictive processing (PP) is based on the idea that biological systems instantiate an internal model of their environment that allows them to make predictions, take actions, and to minimize surprise [14–17]. In the PP formalism, an agent uses internal states to form an internal model of its environment based on perceived stimuli (Fig 1Ai). We focus here on a form of surprise that results from the mismatch between the agent’s internal state and the feedback from the environment through sensory stimuli (see e.g. [18, 19] for an in-depth discussion of different definitions of surprise). In general, these stimuli map only parts of the environment’s true state, implying an unavoidable residual level of uncertainty. To reduce the uncertainty, the agent uses the internal model to formulate predictions about the environment and performs actions to test these predictions. These actions may lead to new stimuli that provide feedback about the environment’s true state, triggering an update of the internal model.

We apply PP to individual synapses, arguing that the dynamics of a synapse can be considered as an agent interacting with its cellular environment, and derive a synaptic learning rule by minimizing the surprise in individual synapses. This learning rule enables synapses to adapt their synaptic efficacy to best predict future postsynaptic spikes, which are registered by back-propagating action potentials (bAPs). In contrast to previous approaches (e.g., [20]) that used PP to understand the influence of neuromodulatory signals such as Dopamine on synaptic plasticity, we focus here on a PP perspective of synaptic plasticity [21, 22], unraveling the dynamics of a single synapse governed by only locally accessible quantities such as the pre- and postsynaptic-spike times and the current value of the synaptic efficacy. This is the only assumption made in our model. The presented synaptic plasticity rule follows directly from this assumed interaction between a synapse and its post-synaptic neuron.

Predictive processing has emerged as a powerful normative theory to derive biologically plausible rules for synaptic plasticity from first principles, and a number of previous studies have explored this, e.g., to address the role of neural compartmentalization [21, 22], cortical mocrocircuits [23], and dendritic trees for few short learning [24]. In the current study we focus on the temporal dynamics of the uncertainty that is being induced by sparse back-propagating action potentials and the role of noise in synaptic transmission. This synapse-centric view on predictive processing allows us to study the precise timing of synaptic dynamics in terms of spike-timing-dependent plasticity.

We call our new model the *synaptic predictive processing* (SPP). The emergent synaptic plasticity rule reproduces a number of experimentally observed effects of long-term potentiation (LTP) and depression (LTD) protocols and predicts precise forms for the influence of synaptic and neuron parameters. The SPP suggests that synapses probe their environment by sending stochastic synaptic currents and integrate the arriving feedback (bAPs) to update their internal state (synaptic efficacy) to better predict the somatic dynamics. Thus, every stochastic release event can be seen as a “*small experiment*”, that is based on previous experience and the outcome of which shapes subsequent future activity. In other words, as we show here, the task of a synapse to learn suitable synaptic responses can be considered as a problem of behaving in a partially unknown environment, where the variability in synaptic release is being used to properly represent the uncertainty of the synapse about the cellular, environmental state. On the network level, our computer simulations indicate that SPP allows several thousand synapses to exploit their synaptic noise to successfully master different learning paradigms despite ambiguous or uncertain inputs.

## Materials and methods

### Neuron model

We used the leaky integrate and fire (LIF) neuron model [25] in all experiments, where the somatic membrane potential *u*(*t*) at time *t* > 0 follows the dynamics

$$\begin{array}{c} \tau_{m}\frac{d\;u}{d\;t}\;=\;-(u\left(t\right)-u_{0})\;+\;R\;y\left(t\right)\;, \end{array}$$ (1)

where *τ*_*m*_ is the membrane time constant, *u*_0_ is the resting potential and *R* the membrane resistance. *y*(*t*) is the external input current into the neuron, and denotes the effect of afferent synaptic input at time *t*. When the membrane potential reaches the threshold *ϑ*, the neuron emits an action potential, such that the spike times *t*_*f*_ are defined as the time points for which the criterion

$$\begin{array}{c} t_{f}\;:\;u(t_{f})=\vartheta \;, \end{array}$$ (2)

applies. Immediately after each spike, the membrane potential is reset to the reset potential *u*_*r*_ [25]

$$\begin{array}{c} \underset{\delta \to 0}{\text{lim}}\;u(t_{f}+\delta )=u_{r}\;, \end{array}$$ (3)

and we define the initial state of the neuron *u*(0) = *u*_*r*_.

In network simulations, we used a simple threshold adaptation mechanism to control the output rate of the neurons. Individual firing thresholds *ϑ* were used for every neuron. If not stated otherwise, thresholds were decreased by a value of $u_{\text{adapt}}^{\left(-\right)}=5\times 10^{-5}$ mV in every millisecond and increased by $u_{\text{adapt}}^{\left(+\right)}=10^{-2}$ mV after every output spike. Thresholds values were clipped from below at the resting potential *u*_0_.

### Synapse model and learning rule

A detailed derivation of the synapse model can be found in Sections 1–8 in S1 Appendix.

We use a stochastic synapse model of input-dependent PSCs, where the variability is proportional to the synaptic efficacy *w* [26]. The amplitudes of current pulses, *y*, were drawn from a Gaussian distribution, $y∼N(y\;|\;\frac{r_{0}}{M}\;w,\frac{s_{0}}{M^{2}}\;w)$, independently for every input spike, where *w* is the synaptic weight and *M* is a scaling parameter. The parameter *s*_0_ of the stochastic PSC model was chosen to be a Gaussian approximation of the Binomial distribution with *s*_0_ = *r*_0_(1 − *r*_0_). The scaling parameter *r*_0_ corresponds to the synaptic release probability [26]. Input currents *y*(*t*) were generated as follows: For every pre-synaptic spike, a sharp current pulse was generated and scaled with the amplitudes, sampled randomly and independently, and injected into the post-synaptic neuron. At all other times, the synaptic inputs *y*(*t*) were 0.

**Fig 2:**
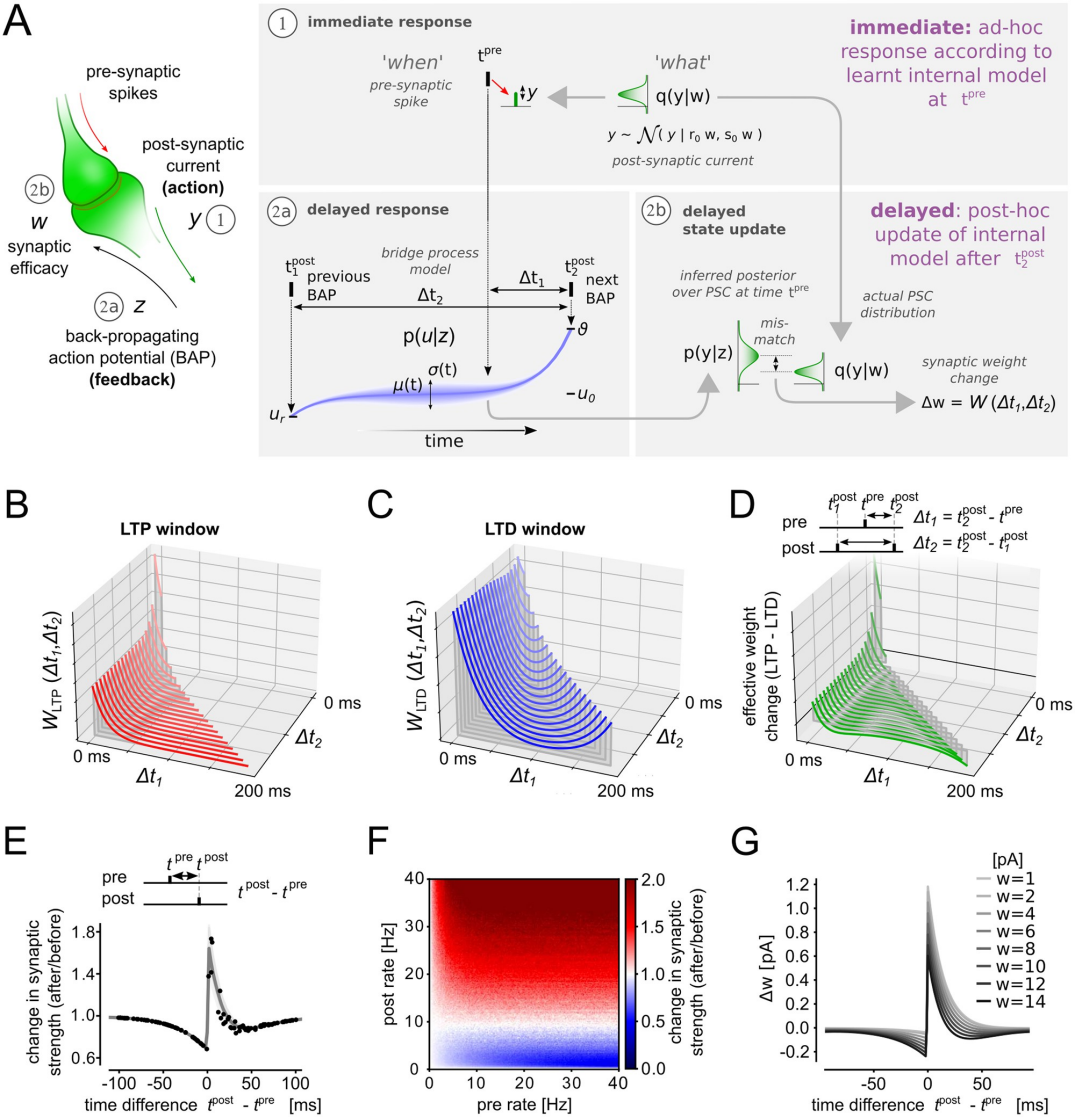
The SPP learning rule resembles regulated triplet STDP. **A:** Illustration of the main steps of the SPP synaptic learning model. **B,C:** The triplet STDP windows *W*_LTP_ (B) and *W*_LTD_ (C) that emerge from SPP as a function of the spike timing differences Δ*t*_1_ and Δ*t*_2_. **D:** The effective synaptic efficacy changes that result from the LTP and LTD windows. **E:** Mean synaptic efficacy changes (gray line) and individual trials (black dots) for an STDP pairing protocol. Shaded area indicates std over trials (Δ*t*_2_ = 500 ms). **F:** synaptic efficacy changes as a function of pre- and post- rate. **G:** Weight dependence of the SPP learning rule plotted as STDP curve as in (E).

If not stated otherwise, the synaptic efficacies *w* were updated using the learning rule,

$$\begin{array}{c} \Delta w\;=\;W_{\text{LTP}}(\Delta t_{1},\Delta t_{2})-(\frac{1}{2}+w)W_{\text{LTD}}(\Delta t_{1},\Delta t_{2})+\frac{1}{2\;w}, \end{array}$$ (4)

where $W_{\text{LTP}}(\Delta t_{1},\Delta t_{2})$ and *W*_LTD_(Δ*t*_1_, Δ*t*_2_) are the triplet STDP windows as depicted in Fig 2. $\Delta t_{1}=t_{2}^{\text{post}}-t^{\text{pre}}$ and $\Delta t_{2}=t_{2}^{\text{post}}-t_{1}^{\text{post}}$ are the relative firing times of neighboring pre- and post-synaptic spikes (see Fig 2A–2D). In Section 8 in S1 Appendix we show in detail that the synaptic efficacy updates Eq (4) minimize the surprise of the back-propagating action potentials *z* with respect to the synaptic efficacy *w*. We further show that the triplet STDP windows can be defined analytically using the stochastic bridge model [27, 28]. This model describes the dynamics of the membrane potential with time varying mean and variance functions *μ*(Δ*t*_1_, Δ*t*_2_) and *σ*^2^(Δ*t*_1_, Δ*t*_2_), respectively:

$$\begin{array}{c} \mu (\Delta t_{1},\Delta t_{2})\;=\;u_{0}\;+\;\left(u_{r}-u_{0}\right)\frac{e^{\frac{\Delta t_{1}}{\tau_{m}}}-e^{-\frac{\Delta t_{1}}{\tau_{m}}}}{e^{\frac{\Delta t_{2}}{\tau_{m}}}-e^{-\frac{\Delta t_{2}}{\tau_{m}}}}\;+\;\left(\vartheta -u_{0}\right)\frac{e^{\frac{\Delta t_{2}-\Delta t_{1}}{\tau_{m}}}-e^{\frac{\Delta t_{1}-\Delta t_{2}}{\tau_{m}}}}{e^{\frac{\Delta t_{2}}{\tau_{m}}}-e^{-\frac{\Delta t_{2}}{\tau_{m}}}}\;, \end{array}$$ (5)

and

$$\begin{array}{c} \sigma^{2}(\Delta t_{1},\Delta t_{2})\;=\;\sigma_{0}^{2}\;\frac{1}{1\;+\;\gamma (e^{\frac{\Delta t_{1}-\Delta t_{2}}{\tau_{m}}}+e^{-\frac{\Delta t_{1}}{\tau_{m}}})}\;, \end{array}$$ (6)

where *σ*_0_ and *γ* are synaptic constants that scale the noise contribution to *u*.

### Numerical simulations

All simulations were performed using a custom implementation that was developed using Python (3.8.5) and can be found using the following link: https://gitlab.com/kappeld/synaptic-predictive-processing. We used the Euler method to approximate the solution of the stochastic differential equations with a fixed time step of 1 ms. Postsynaptic currents were created as described in Section 3 in S1 Appendix, where Dirac delta pulses were approximated by 1 ms rectangular unit pulses. Current pulses were truncated at zero. If not stated otherwise, we used a synaptic release parameter $r_{0}=\frac{1}{2}$. In Eq (1) the membrane time constant *τ*_*m*_ was 30 ms, the resting potential *u*_0_ was -70 mV and the membrane resistance *R* was 40 M*Ω*. The firing threshold *ϑ* was -55 mV, *u*_*r*_ was -75 mV and the learning rate *η* was 5 × 10^−4^. In Eq (6) we chose $\sigma_{0}^{2}=16$ and *γ* = 50 by hand, to approximately match the typical variability of the membrane potential (see Fig 2B and 2C for an illustration). Weights were drawn randomly and independently from a Gaussian distribution with mean and standard deviation of *w*_*init*_ = 10 pA and clipped at 0 before learning. See Table 1 for a summary of the simulation parameters.

**Table 1:** Default parameter values used throughout the simulations.

Parameter	Value	Description
Δ*t*	1 ms	simulation time step
*u*_0_	-70 mV	resting potential
*u*_*r*_	-75 mV	reset potential
*ϑ*	-55 mV	firing threshold (or initial value if adaptive)
*τ*_*m*_	30 ms	membrane time constant
*R*	40 M*Ω*	membrane resistance
*r*_0_	$\frac{1}{2}$	release probability
*N*_hidden_	25	number of hidden neurons
*N*_input_	100	number of input channels
*w*_*IE*_	-5 pA	inhibitory to excitatory weight
*w*_*init*_	10 pA	mean and std of initial excitatory weights
*η*	5 × 10^−4^	learning rate

In Fig 2, we used a single SPP synapse, applied different pre/post spike pairing protocols and recorded the resulting weight changes. In Fig 2E, STDP spike protocols were repeated 50 times with a time delay of 500 ms between two pairings (fixed Δ*t*_2_ = 500 ms). In Fig 2F, independent Poisson spike trains were presented to the synapses for 100 s. Fig 2G the STDP protocol from Fig 2E was repeated with different initial weights.

**Fig 3:**
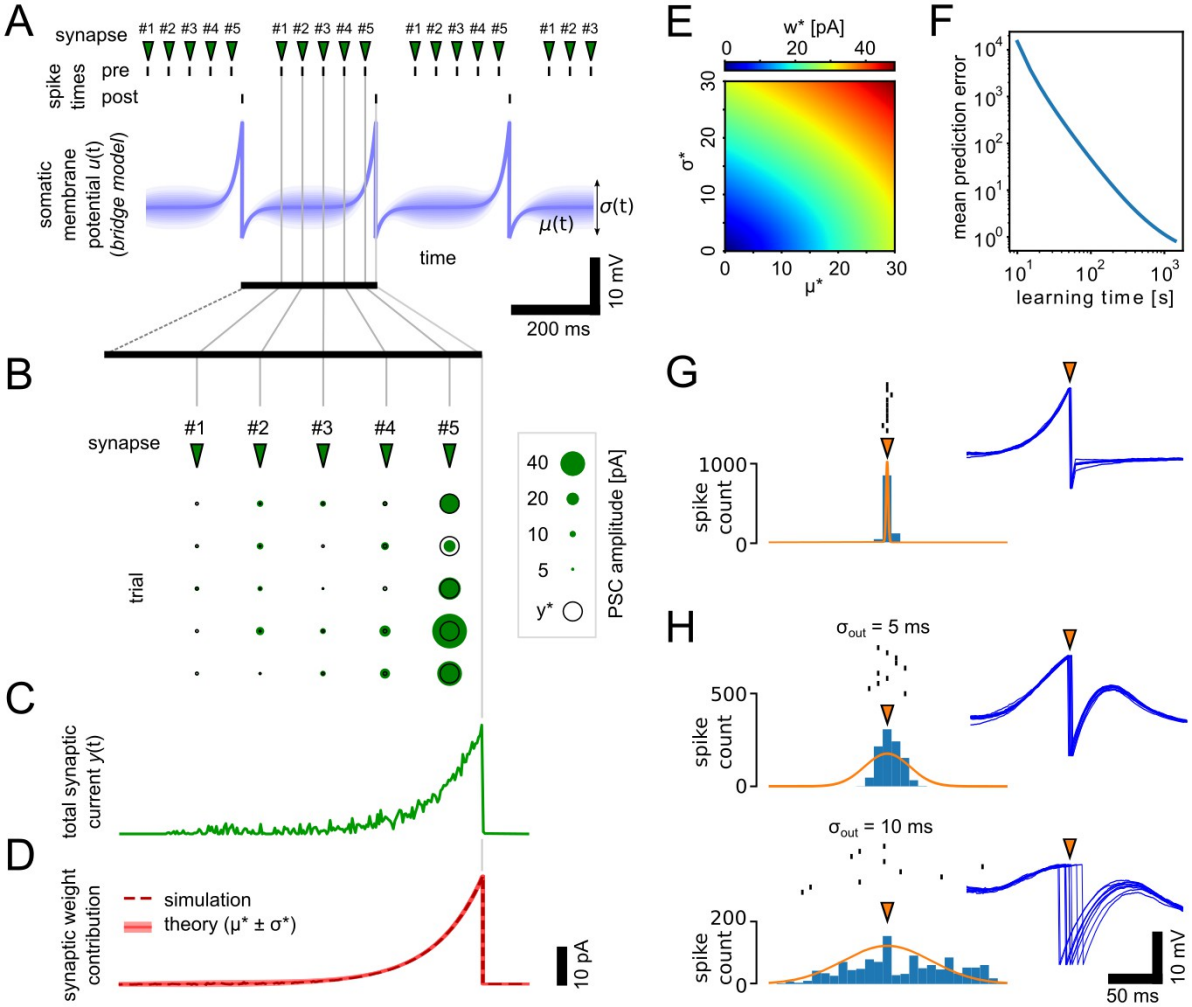
Synapse-level probability matching. **A:** *μ*(*t*) and *σ*(*t*) of the somatic membrane potential given the stochastic bridge model for a neuron that is brought to fire with a spike interval of 300 ms. Pre-synaptic neurons were brought to fire at fixed time offsets relative to the post-synaptic spikes. **B:** Synapses learn to inject the optimal current that matches the bridge model in (A). Empty circles indicate the theoretically optimal currents y*. Individual current pulses are shown for multiple trials for synapses with different time offsets. **C:** The combined effect of all synapses shown by the summed input current for a single trial. **D:** Synaptic efficacies after learning and weight means and stds predicted by the theory. **E:** Synaptic efficacies after learning are correlated with the theoretically derived μ* and σ* (see panel D). **F:** The mean prediction error over all synapses declines throughout learning. **G:** Firing behavior of the neuron after learning when allowed to fire freely in response to input spikes. 10 individual spike times are shown together with histograms over 1000 trials. Insets show membrane dynamics during the 10 trial runs. The orange arrow indicates spike time during learning. **H:** As in (G) but here the output spike times were given by Gaussian distributions of different spreads. The orange arrow indicates here the mean.

In Fig 3, we used single neurons that received input from 300 afferent input neurons. Input neurons fired a dense syn-fire chain where every neuron was emitting a spike in exactly 1 ms during a 300 ms time window. The post-synaptic neuron was brought to fire at the end of this pattern. In Fig 3F and 3G, the output firing was determined by the intrinsic neuron dynamics after learning. In Fig 3G output spike times were drawn from a Gaussian distribution with different standard deviations during learning. The prediction error in Fig 3F was computed as the Kullback-Leibler divergence $D_{\text{KL}}(q(y\;|\;w)\;∥\;p(y\;|\;z))$ (see Section 8 in S1 Appendix for details).

**Fig 4:**
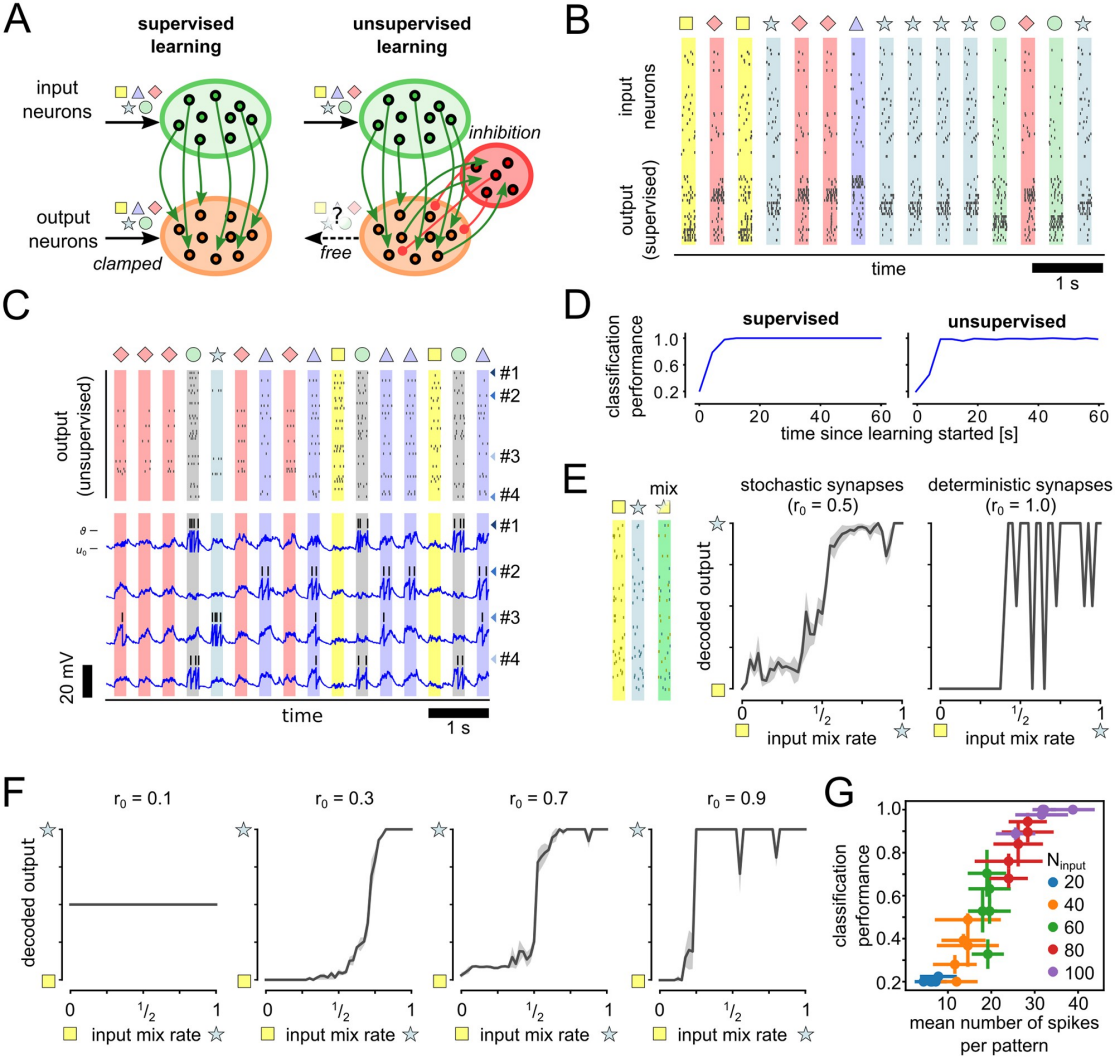
SPP learning rule for supervised and unsupervised learning scenarios. **A:** Illustration of the network structure with synapses shown in green being adapted by SPP. Five independent spike patterns (▫, ☆, △, ◇, ○) are presented to the network via the input neurons. Output neurons are either clamped to pattern-specific activity during learning (supervised) or allowed to run freely (unsupervised). **B:** Learning result using the SPP rule for the supervised scenario. Typical spiking activity of the network after learning for 60 s. Black ticks show output spike times. **C:** Output activity after learning for the unsupervised scenario. Traces of membrane potentials are shown for selected output neurons (matching color-coded arrows indicate neuron identities). **D:** Classification performance for supervised and unsupervised learning scenario. Classification performance plateaus at near optimal value after about 20 s of learning time for both supervised and unsupervised scenario. **E:** Spike patterns of two input symbols (▫, ☆,) were mixed with different mixing rates (example pattern shows mixing rate 1/2). Uncertainty is reflected in output decoding (left) if inputs are ambiguous (around mixing rate of 1/2). If synapse noise is disabled (*r*_0_ = 1), uncertainty is not represented in the output (right). Mean and std over classification scores of 5 input pattern presentations are shown. **F:** As in (E) but for different levels of release probability *r*_0_. **G:** Classification performance for different number of input neurons. Error bars show mean and std.

In Fig 4, spike patterns were generated by randomly drawing values from a beta distribution (*α* = 0.2, *β* = 0.8) for each input channel and multiplying these values with the maximum rate of 20 Hz. From these rate patterns individual Poisson spike trains were drawn for every pattern. During the learning phase, the output neurons were clamped to fire 50 Hz Poisson spike trains during presentation of the preferred stimulus pattern and remain silent otherwise. Pattern presentations were interleaved with phases of 200 ms of zero spiking on all input channels. In the supervised scenario, for every output neuron one of the five patterns was selected as preferred stimulus. During training, the activity of the output neurons was clamped to fire during the presentation of the preferred stimulus pattern.

The following evaluation methods were used to assess the classification performance. Network output spikes were recorded during the presentation of sample input spike patterns. Output spikes from neurons with the same preferred stimulus were combined into groups for this analysis. If the neuron group with the largest number of spikes was the same as the group associated with the presented input pattern, the score was 1. If the maximum was not unique, i.e., if *G* groups produced exactly the same number of spikes, the score was $\frac{1}{G}$. Otherwise, the score was 0. Unless otherwise stated, mean and std of scores over 5 pattern presentations were reported to evaluate classification performance.

In the unsupervised scenario, the network was augmented with fixed lateral inhibition to stabilize the firing behavior during learning. All excitatory neurons were connected to the inhibitory neuron with a synaptic efficacy of 1. The inhibitory unit projected back to all excitatory neurons with a synaptic efficacy of -5. During learning, output neurons were allowed to run freely according to their intrinsic dynamics. The SPP learning rule was used at all synapses between input and output neurons in both scenarios. In Fig 4E we set the synaptic release parameter *r*_0_ to 1 to disable synaptic noise (‘*without noise*’ condition).

The mixed patterns in Fig 4E were generated by randomly swapping spikes between two fixed spike patterns. The number of spikes to swap was proportional to the input mixing rate. The spike permutation for swapping was kept fixed throughout the experiment in Fig 4E so that no input noise was introduced by pattern mixing.

To evaluate the unsupervised classification performance in Fig 4D, we trained a linear classifier on the network outputs. To do this, we generated an independent training set by sampling the network activity after training from the network output layer. The output spikes were then binned according to the time windows of the input pattern presentations to generate a spike count per neuron and pattern presentation. The spike counts were then used to train a linear support vector machine (SVM) with L2 regularization, using the pattern identities as targets. The reported classification performance was then evaluated on a separate dataset using the trained network and SVM.

**Fig 5:**
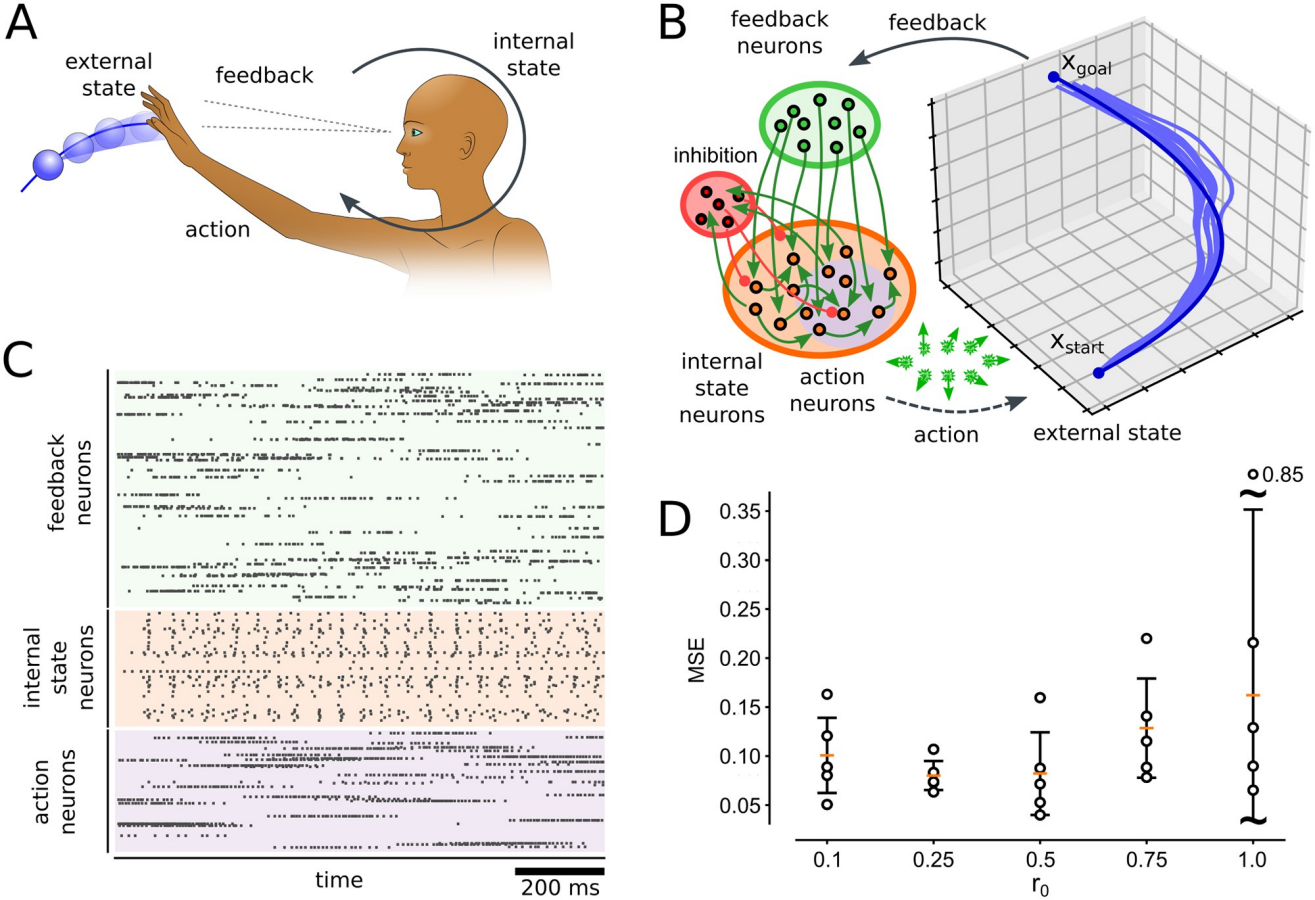
The SPP for learning a closed-loop behavior in a recurrent network. **A:** Illustration of the behavior level PP for an agent that interacts with a dynamic environment. **B:** A spiking neural network interacting with an environment using SPP to learn a control policy. The activity of action neurons controls the movement of an agent in a 3-dimensional environment. Feedback about the position of the agent is provided through feedback neurons. The policy to navigate the agent is learned through SPP between feedback and action neurons. The training trajectory (dark blue) and 8 spontaneous movement trajectories generated by the network after learning (light blue) are shown. Action neuron preferred directions indicated in green (not to scale). **C:** Spike train generated spontaneously by the network after learning corresponding to one movement trajectory in (B). **D:** Learning performance (MSE) for different release probability parameters *r*_0_. Bars indicate mean and std over five independent runs.

In Fig 5 we used a recurrent network with 400 feedback neurons and 400 internal state neurons from which we selected 200 action neurons. Preferred positions of the feedback neurons were scattered uniformly over the action space, and firing rates were scaled by Gaussian tuning curves between agent and preferred position, with variance parameter 5 mm. Action neurons preferred direction were randomly assigned to preferred movement directions sampled from a sphere with diameter 2.5 mm. The environment was a 3D Euclidean space. Start and goal location were placed at random locations at a distance of around 2.7 m. Internal state neurons received lateral inhibitory feedback and rate adaptation similar as in Fig 4. Here, we used a faster adaptaion mechanism with $u_{\text{adapt}}^{\left(-\right)}=2\times 10^{-4}\;\text{mV}$ and $u_{\text{adapt}}^{\left(+\right)}=4\times 10^{-2}\;\text{mV}$. During spontaneous movement, the agent’s end effector was set to *x*_*start*_ at trial onset, and then the position was updated every 50 ms by adding the decoded position offset provided by the action neurons (light blue traces in Fig 5). During training, the activity of action neurons was clamped according to a pre-defined training trajectory (dark blue in Fig 5) of duration 1.2 s.

## Results

After introducing the reasoning to link synaptic properties with the predictive processing and the fundamentals of the considered model, we sketch the derivation of the resulting synaptic plasticity rule and compare it with experimental data. We show that the plasticity rule of SPP coordinates the unreliability in synaptic transmission of a group of synapses to drive their joint postsynaptic neuron selectively. This successful coordination also functions in feedforward as well as recurrent neuronal networks, allowing the system to decode reliably ambiguous stimuli or to behave successfully in dynamic environments.

### A synaptic account of predictive processing

The PP framework provides a generic approach to model the behavior of an agent that interacts with its environment. The main assumption is that the agent and the environment have separated states, and the PP framework provides a formalism to describe the interaction between these states. Interaction between the agent’s (*internal*) and the environment’s (*external*) state only takes place through *actions* performed by the agent and *sensory feedback* provided by the environment (see Fig 1Ai). The *internal state* → *action* → *external state* → *feedback*—loop eventually causes a mismatch between sensory feedback and the agent’s internal state that is described here as surprise. This renders an optimization problem with the goal of minimizing surprise, that can be solved by maintaining an internal model of the environment, allowing the agent to reason about the true external state and its own uncertainty about it. Sensory feedback is used to update the internal model and state of the agent such that future actions better help the agent to predict the environment.

The intuition behind our synapse-centric SPP model is illustrated in Fig 1Aiii. We consider the synapse as an agent and the postsynaptic neuron as its environment. Furthermore, we consider the somatic membrane potential *u* as the state variable of the neuron, since it determines the neuron’s spiking behavior. However, as suggested by experimental findings [29], the actual value of the somatic membrane potential is hidden from the synapse and therefore the synapse has to infer the value of *u* from the sparse information that propagates back from the soma into the dendrites. We consider that this sparse feedback is implemented by bAP events *z*, given by the firing times $t_{1}^{\text{post}},t_{2}^{\text{post}},\ldots ,t_{n}^{\text{post}},\ldots$ of the postsynaptic neuron. We model *z* as Dirac delta pulses placed at the bAP event times, i.e. $z\left(t\right)=\sum_{n}\;\delta (t_{n}^{\text{post}}-t)$. For simplicity, we neglect propagation delays between soma and synapse.

Thus, in this model, the synapse only receives information about the value of the somatic membrane potential *u* at the post-synaptic firing times. This information denotes whether the somatic membrane potential has recently reached the firing threshold (if $u(t=t_{n}^{\text{post}})=\vartheta$, then $z(t=t_{n}^{\text{post}})>0$) or not (if *u*(*t*) < *ϑ*, then *z*(*t*) = 0), but does otherwise not contain information about the true value of *u*. The feedback thus causes surprise that is being incorporated into the update of the internal model to better guide the agent’s actions. We model the surprise as the negative log likelihood,

$$\begin{array}{c} \text{surprise}\left(z\right)\;=\;-\text{log}\;p(z)\;. \end{array}$$ (7)

The interrelations between synapses and soma imply in the SPP model that bAPs *z* can trigger an update of the synapse’s internal state, yielding synaptic plasticity.

In summary, by employing PP on an individual synapse, we find that the exchange of information between agent and environment are determined by spikes, which are in general very sparse, such that sensory feedback and actions only happen at specific time points. This suggests an event-based view on PP where actions (and feedback) are only provided at certain triggering times (Fig 1Aii). This view allows us to separate the ‘*what*’ and ‘*when*’ information flow in the synapse model, which leads to local synaptic weight updates (see derivation in Sections 1–8 in S1 Appendix).

The actions of a synapse to interact with the soma are given by the postsynaptic currents (PSCs) *y* (‘*what*’) that are released in response to pre-synaptic spikes (‘*when*’). In other words, in the SPP the pre-synaptic spikes operate as a trigger for the synapse to initialize an action implemented by PSCs (Fig 1Aii and 1Aiii, red arrow). For simplicity we consider the PSC generation as a process of the whole synapse including pre- and postsynaptic mechanisms. Although individual synaptic mechanisms can have specific noise properties [1, 30], we integrate pre- and postsynaptic noise sources into one Gaussian noise source that influences the amplitude of PSCs (see Section 2 in S1 Appendix for details). Thus, at pre-synaptic spike times *t* = *t*^pre^ PSCs are drawn from a general normal distribution with mean and variance being scaled by the synaptic efficacy *w*, in accordance with experimental findings [1, 4, 30].

$$\begin{array}{c} y\left(t\right)=\sum_{m}\delta (t_{m}^{\text{pre}}-t)\;y_{m},\;\text{with}\;y_{m}∼q(y_{m}\;|\;w)\;=\;N(y_{m}\;|\;\frac{r_{0}w}{M},\frac{s_{0}w}{M^{2}}), \end{array}$$ (8)

where *r*_0_ > 0 and *s*_0_ > 0 are constants that scale the mean and variance of synaptic currents, related to the synaptic release probability, and *M* is a constant that scales the PSC amplitudes overall. *q*(*y* | *w*) determines the distribution over PSC amplitudes for a given synaptic internal state *w*. At all other times the PSC equals zero (see Fig 1D).

To describe the relationship between the feedback and the true external state, the agent maintains an internal model of the environment. As the feedback is sparse in time and information (see above), the internal model makes use of a probability distribution over the likelihood of environmental states. This implies that the agent keeps track of its uncertainty about the true external state. In general, the environment is too complex to directly infer the external state given the feedback in terms of a posterior probability distribution [31]. However, we find that for the SPP we can express this distribution *p*(*u* | *z*) directly in closed form by considering the so-called stochastic bridge model [27] as “synaptic” approximation of the somatic membrane dynamics *u*(*t*) (see Section 4 in S1 Appendix for more details).

To do so, we have to remind ourselves of the fact that a bAP at a time $t=t_{1}^{\text{post}}$ conveys the information to a synapse that the postsynaptic, somatic membrane potential has just reached the firing threshold $u(t=t_{1}^{\text{post}})=\vartheta$. To describe the membrane dynamics between any two bAPs at $t_{1}^{\text{post}}$ and $t_{2}^{\text{post}}$, we can utilize that the synaptic uncertainty about the true membrane potential is minimal close to the spike times and maximal between both spikes at $\frac{t_{2}^{\text{post}}-t_{1}^{\text{post}}}{2}$. Such an event-based time course of the uncertainty is captured by the stochastic bridge model that determines the distribution of *u*(*t*) given *z* through time-varying mean and variance functions *μ*(*t*) and *σ*^2^(*t*), respectively. Importantly, the shape of *μ*(*t*) and *σ*^2^(*t*) between any pair of postsynaptic spikes depends only on the interspike interval $t_{2}^{\text{post}}-t_{1}^{\text{post}}$. Fig 1C shows the solution of the stochastic bridge model for $t_{2}^{\text{post}}-t_{1}^{\text{post}}$ = 100 ms that sufficiently captures real membrane dynamics (see Fig 1B for one example). In other words, we can use the stochastic bridge model to describe the internal representation that the synapse maintains about the somatic membrane dynamics. In the next section, we will show that the stochastic bridge model can also be used to infer biologically plausible synaptic plasticity rules to adapt the synaptic efficacies *w*, and that this implies an update of the internal model of the synapse.

### Synaptic plasticity as surprise minimization

Learning in the SPP means to adapt the synaptic efficacy *w* to minimize the “surprise” caused by bAPs *z*, where surprise is measured with respect to the synapse’s internal model of the soma *p*(*u* | *z*). In other words, the feedback *z* triggers an update of the internal state of the synapse *w*. This update changes the actions of the synapse, namely the PSC amplitudes *y*. The changed actions in turn adapt the dynamics of the somatic membrane potential and, thus, the firing of the postsynaptic neuron that is fed back to the synapse by bAPs *z*. To better understand this loop, we split the effect of the synaptic efficacy into (1) an immediate and (2) a delayed response that are triggered by pre- and post-synaptic firing. Using the event-based view of the SPP (Fig 1Aii), each of these responses can be divided into a “*when*” and a “*what*” part. Both responses together determine the adaptation of the synaptic efficacy. The complete process of the synaptic efficacy update Δ*w* is illustrated in Fig 2A.

The immediate response (1) determines the action *y* of the synapse. At the time of pre-synaptic firing *t*^pre^ (“*when*”) a PSC amplitude is generated by drawing a value for *y* from the distribution *q*(*y*|*w*) given in Eq (8) (“*what*”). The synaptic efficacy *w* determines both, the mean and variance of *y*. The mathematical formulation could be extended comprising two parameters that determine mean and variance separately, but this is not investigated here. The immediate response *y*, in other words, constitutes an ad-hoc guess about the firing behavior of the postsynaptic neuron, based on past experience encoded in *w*, before more information is provided by further bAPs (*z*).

The actual update of the synaptic efficacy happens during the delayed response (2). After a new bAP has arrived, the internal model *p*(*u* | *z*) is used to update *w* such that the distribution *q*(*y* | *w*) (immediate response) better reflects (or predicts) the postsynaptic firing behavior. The “*when*” part of this update is determined by pre- and post-synaptic firing times. Hereby, the internal model *p*(*u* | *z*) is used to align the relative timing of pre- and post-synaptic firing. This information is then used to estimate the distribution over values of *u* (“*what*”) from which the weight update is inferred. The delayed response thus constitutes a post-hoc correction of the PSC probability distribution *q*(*y* | *w*).

To understand the actual weight update mathematically, we have to divide the delayed response (2) into two sub-problems: (2a) We have to invert the internal model *p*(*u* | *z*) to obtain a posterior distribution *p*(*y* | *z*) over synaptic currents *y* that most likely lead to a desired spiking behavior (measured by *z*). (2b) Then we have to reduce the surprise Eq (7) by reducing the distance between the inferred posterior distribution *p*(*y* | *z*) and the actual distribution over PSCs *q*(*y*|*w*) used in the immediate response (1) to update the synaptic efficacy *w*.

To solve the first sub-problem (2a) we make use of the internal model *p*(*u* | *z*) to directly infer PSCs that are compatible with a given spiking behavior *z*. In Section 4 in S1 Appendix we show that *p*(*y* | *z*) can be analytically expressed using the stochastic bridge model to describe *p*(*u* | *z*). The resulting solution of the posterior distribution is given by a Gaussian distribution with time-varying mean and variance function *m* and *v*, respectively. At any time *t* = *t*^pre^ the posterior over *y* can thus be written as

$$\begin{array}{c} p(y\;|\;z)\;=\;N(y\;|\;m(\Delta t_{1},\Delta t_{2}),v(\Delta t_{1},\Delta t_{2}))\;, \end{array}$$ (9)

where $\Delta t_{1}=t_{2}^{\text{post}}-t^{\text{pre}}$ and $\Delta t_{2}=t_{2}^{\text{post}}-t_{1}^{\text{post}}$ are the relative firing times. This distribution has the property, that it generates PSCs *y* that will, with high probability, result in a spiking behavior *z* when injected into the post-synaptic neuron. Importantly, the functions *m* and *v* only depend on Δ*t*_1_, Δ*t*_2_.

The posterior PSC distribution *m*(Δ*t*_1_, Δ*t*_2_) and *v*(Δ*t*_1_, Δ*t*_2_) in Eq (9) were computed for the LIF neuron model (Eq 1), and are given by

$$\begin{array}{c} \begin{array}{cc} m(\Delta t_{1},\Delta t_{2}) & \;=\;\mu^{\prime }\left(\Delta t_{1},\Delta t_{2}\right)+\frac{1}{\tau_{m}}(\mu \left(\Delta t_{1},\Delta t_{2}\right)-u_{0})\;,\\ v(\Delta t_{1},\Delta t_{2}) & \;=\;{\left(\sigma^{2}\left(\Delta t_{1},\Delta t_{2}\right)\right)}^{\prime }+\frac{2}{\tau_{m}}\sigma^{2}\left(\Delta t_{1},\Delta t_{2}\right)\;, \end{array} \end{array}$$ (10)

where $f^{\prime }\left(t\right)=\frac{d}{dt}f\left(t\right)$ denotes the time derivative (see Section 6 in S1 Appendix for a detailed derivation).

To solve sub-problem (2b) in Fig 2A, i.e. to minimize the surprise of observing *z* with respect to the synaptic efficacy *w*, we can use Eqs (8) and (9) to directly minimize the distance D(q|p) between *q*(*y*|*w*) and *p*(*y* | *z*). To do so it is sufficient to consider the relative firing times $\Delta t_{1}=t_{2}^{\text{post}}-t^{\text{pre}}$ and $\Delta t_{2}=t_{2}^{\text{post}}-t_{1}^{\text{post}}$ (see Fig 2A–2D). Hereby, Δ*t*_1_ and Δ*t*_2_ can be linked to learning windows of spike-timing-dependent plasticity (STDP, Fig 2B and 2C). These learning windows implicitly encode the relevant dynamics of the stochastic bridge model, and thus the internal model does not have to be encoded explicitly in every synapse. The synaptic efficacy updates can then be expressed in the form (see Section 8 in S1 Appendix for a detailed derivation)

$$\begin{array}{c} \Delta w\;=\;-\frac{\partial }{\partial w}D\left(q|p\right)\;=\;W_{\text{LTP}}(\Delta t_{1},\Delta t_{2})-(\frac{1}{2}+w)W_{\text{LTD}}(\Delta t_{1},\Delta t_{2})+\frac{1}{2\;w} \end{array}$$ (11)

where *W*_LTP_(Δ*t*_1_, Δ*t*_2_) ≥ 0 and *W*_LTD_(Δ*t*_1_, Δ*t*_2_) ≥ 0 are triplet STDP learning windows that depend only on the relative timing Δ*t*_1_ and Δ*t*_2_ of pre- and post-synaptic firing, and where *w* denotes the current value of the synaptic efficacy.

In summary, the main required steps to compute the synaptic weight updates according to the SPP model (Fig 2A), are the following: The arrival of a pre-synaptic spike at time *t*^pre^ leads to an ad-hoc response by generating a postsynaptic current *y* according to the internal model *q*(*y*|*w*). When the next bAP arrives at the synapse, a post-hoc update of the synaptic efficacy *w* is triggered according to Eq (11). The probabilistic model Eq (9) does not have to be explicitly represented in the synapse but can be implicitly represented by the shape of the learning windows *W*_LTP_(Δ*t*_1_, Δ*t*_2_) and *W*_LTD_(Δ*t*_1_, Δ*t*_2_).

The functional form of the two triplet STDP windows is determined by the neuron dynamics (Fig 1C), and depend on Δ*t*_1_ and Δ*t*_2_ in a nonlinear manner [32]. Using Eq (9) the STDP windows can be expressed as

$$\begin{array}{c} W_{\text{LTP}}(\Delta t_{1},\Delta t_{2})=r_{0}\;\frac{m(\Delta t_{1},\Delta t_{2})}{v(\Delta t_{1},\Delta t_{2})}\;\text{and}\;W_{\text{LTD}}(\Delta t_{1},\Delta t_{2})=r_{0}^{2}\;\frac{1}{v(\Delta t_{1},\Delta t_{2})}\;. \end{array}$$ (12)

The PSC variance (*v*) has a divisive contribution to both STDP windows. Thus, the learning windows have large values where the uncertainty about *y* is lowest (small values for *v*). In Fig 2B–2D we plot the learning windows for different values of Δ*t*_1_ and Δ*t*_2_. *W*_LTP_ has a potentiating effect which is maximal close to Δ*t*_1_ = 0 (Fig 2B). This is a manifestation of Hebbian-type learning, where close correlations of pre- before post- firing leads to potentiation. *W*_LTD_ is a depression term (Fig 2C). Both STDP windows show also a strong rate dependence (Δ*t*_2_) as higher firing rates result in less uncertainty about *u*(*t*). Fig 2D shows the combined effect of the LTP and LTD.

In Fig 2E we study an STDP pairing protocol where single pre-/post spike pairs with different time lags Δ*t* were presented to a model synapse [32]. The resulting synaptic changes with respect to Δ*t* = *t*^post^ − *t*^pre^ closely match experimentally measured STDP windows [33, 34]. In Fig 2F we further study the rate dependence of the SPP learning rule. Random pre- and post-synaptic Poisson spike trains were generated with different rates. The learning rule Eq (11) shows a strong dependence on the post-synaptic firing rate. For low pre- or post-synaptic rates synaptic efficacy changes were zero, moderate post-synaptic rates lead to LTD, whereas high post-synaptic rates manifested in pronounced LTP. This rates-weight-change relation is consistent with previous models of calcium-based plasticity [35].

The learning rule Eq (11) also includes a dependence on the current synaptic efficacy to regulate the synaptic strength to not grow out of bounds (cf. [36, 37]). The last term $\frac{1}{2w}$ becomes effective when the synaptic efficacy shrinks to values close to zero and prevents the synaptic efficacy from becoming negative (negative weights have no meaning in our model as they also encode variances). In Section 8 in S1 Appendix we show the mathematical derivation of the learning rule Eq (11) and that *w* ≥ 0 holds for all synapses being initialized with a positive weight value. The weight dependence of the LTD learning window increases the influence of depression for larger synaptic efficacies. In Fig 2G we further analyze the weight dependence of the learning rule. STDP protocols for synapses with different initial synaptic efficacies were applied. Small synaptic efficacies (*w* = 1 pA) lead to learning windows that are positive for all lags Δ*t* (LTP only). Large synaptic efficacies *w* = 12 pA lead to pronounced LTD.

In summary, the SPP learning rule contains features of Hebbian learning, STDP and rate-dependent synaptic plasticity to update the synaptic actions (PSC amplitudes) to better predict the somatic membrane dynamics. The learning rule can be described by a post-pre-post triplet STDP rule [32, 38, 39], plus the weight-dependent last term to regulate the parameter range of *w*. Also, the strength of LTD increases with the synaptic efficacy *w*, which gives rise to a homeostatic effect that prevents synapses from growing out of bounds.

### Synapse-level probability matching of firing times

Next, we show how the learned behavior of synapses influences the firing dynamics of the post-synaptic neuron. After the synapse has formed a model of the post-synaptic membrane potential (the environment in PP parlance), it can be used to reproduce state trajectories that match the learned behavior. For the SPP, this means that synapses install a particular firing pattern *z* through synaptic plasticity. To demonstrate this behavior, we consider here a simple example where a single postsynaptic and many pre-synaptic neurons are repeatedly brought to fire at different fixed offset times (five example pre-synaptic neurons illustrated in Fig 3A, top). According to the stochastic bridge model, the membrane potential of the postsynaptic neuron evolves according the mean and variance functions illustrated in Fig 3A, bottom. We forced all neurons to repeatedly fire according to the fixed pattern while the learning rule Eq (11) was active. SPP learning installs behavior in the synapses that supports (or predicts) the neuron dynamics (Fig 3B–3D). Individual PSCs after learning are shown for five example synapses in Fig 3B. The injected currents show high trial-by-trial variability, and the amplitude strongly depends on the relative pre- and post-synaptic firing. Despite these variabilities, the summed effect of all PSCs show a clear increasing trend towards the postsynaptic spike (single trial shown in Fig 3C). In this example, the optimal solutions of synaptic efficacies can be solved analytically. Fig 3D shows the theoretical and simulation results after learning. The synaptic efficacies learn single parameter distributions that encode the theoretically derived μ* and σ*. This is further studied in Fig 3E, where we plot the synaptic efficacies after learning for different values for μ* and σ*. As can be seen w* encodes μ* and σ*, but collapsed into a single value. For μ* = σ* the single parameter model is exact (see Section 8 in S1 Appendix for additional details, including an analytical derivation of the fixed-point solution of the SPP learning rule). Fig 3F shows the estimated mean surprise throughout learning. The surprise steadily declines with learning time.

Fig 3G shows the spiking behavior after learning when the post-synaptic neuron was allowed to fire freely in response to the same input spikes that were used during learning. The firing was strongly aligned with the target activity (trial-based variance of firing times was 0.1 ms). Despite their highly stochastic nature (Fig 3B and 3C) synapses have learned to drive the post-synaptic neuron to fire reliably. The synaptic variability can also be exploited to reflect uncertainty in neural firing. We let the postsynaptic neuron learn to fire according to Gaussian distributions of firing times with different spreads *σ*_*out*_ (Fig 3H, *σ*_*out*_ = 5 ms and *σ*_*out*_ = 10 ms). The variability in firing times is reflected in the neuron spiking after learning. During the phase of stochastic firing we observe a high trial-to-trial variability in the dynamics of the membrane potential (insets in Fig 3H). Note that the pre-synaptic spike times and the LIF neuron model are deterministic here, so the required trial-by-trial variability is generated exclusively by the synapses. Hence, synapses have learned to utilize their intrinsic variability or noise to drive the deterministic neuron to fire according to a defined probability. The variability in the learning task was also reflected in the distribution of synaptic weights. Weights were overall less sparse and weight distributions were wider, for variable outputs (e.g. *w* ∼ 15.7 ± 27.8, mean ± std, for *σ*_*out*_ = 10 ms) than for learning exact output spikte times (*w* ∼ 5.9 ± 11.7).

### Network-level learning using the SPP learning rule

The SPP learning rule lends itself to solve supervised and unsupervised learning scenarios on the level of neuronal networks (see Section 9 in S1 Appendix for a theoretical treatment). To demonstrate this we consider a pattern classification task (Fig 4A). The network consists of input neurons that project to a set of output neurons. In the unsupervised case, we added inhibitory feedback to control the overall firing rate of the network. We generated five spike patterns of 200 ms duration (denoted in Fig 4 by ▫, ☆, △, ◇ and ○) which were used to control the activity of the input neurons.

Fig 4B shows the typical network activity after learning for the supervised scenario. The output neurons reliably responded to their preferred pattern. The output neurons had also learned a sparse representation of the input patterns in the unsupervised case (Fig 4C). Most neurons (46/50) were active during exactly one of the input patterns (e.g. neuron #1 and #2 in Fig 4C, were ○ and △-selective, respectively). Only few neurons (4/50) showed mixed selectivity and thus got activated by multiple stimulus patterns (see neuron #3 and #4 in Fig 4C).

Fig 4D shows the evolution of the classification performance throughout learning. We used a linear classifier on the network output to recover pattern identities. After learning for 60 s the pattern identity could be recovered by a linear classifier with 100% and 98.8% reliability for the supervised and unsupervised case, respectively (see Fig 4D). The necessary competition between neurons to facilitate this firing pattern is provided in our model by lateral inhibition. In addition, the learning rule reinforces bursts as shown in Fig 2, which has the additional effect of favoring one or a few patterns per neuron. These results demonstrate that the SPP learning rule can be applied to supervised learning scenarios and also leads to self-organization of meaningful representations in an unsupervised learning scenario.

To demonstrate the role of stochastic synapitc release in the pattern classification task, we created ambiguous patterns by mixing the spikes of two patterns (▫ and ☆) with different mixing rates (Fig 4E). Mixing rates of 0 (1) corresponds to a pattern that is identical to ▫ (☆). This can be encoded by a network with unreliable (left, noisy synapses) and with reliable synaptic transmission (right, without noise). However, intermediate values of the mixing rate result in high levels of ambiguity that are represented in the neural output of the network with unreliable synaptic transmission by high trial-by-trial variability, but not with noise-free synapses.

In Fig 4F we further analyze the effect of the release probability *r*_0_. Networks were trained with different values of *r*_0_ (0.1, 0.3, 0.7, 0.9) and the output decodings for mixed patterns were evaluated as in Fig 4E. For too low a probability (0.1), the network did not produce any output spikes, was not able to learn the patterns and the output decodings were consequently at chance level. Intermediate values of 0.3 and 0.7 resulted in good learning performance, but the networks were not able to represent uncertainties around the mixing rate of 1/2 well (the case *r*_0_ = 0.5 has the highest trial-by-trial variance). Uncertainty representation was further degraded when *r*_0_ was increased to 0.9. In summary, we found that a release probability around the maximum variance value of 0.5 resulted in the best representation of uncertainty.

In Fig 4G we study the effect of the number of spikes in the input patterns on the behavior of the network. The theoretical derivation of the learning rule assumes a large number of input spikes (*M* → ∞). We were wondering if this assumption prevents the network from functioning if sparse input patterns are used. To test this, we repeated the above training experiment for *r*_0_ = 0.5 using networks with different numbers of input neurons. To evaluate the behavior of the network, we measure the classification performance of the network (1.0 = perfect, 0.2 = chance level). As expected, the performance is strongly correlated with the number of input spikes. However, perfect performance can be achieved with a relatively small number of spikes per pattern of about 32. This shows that despite the theoretical assumption of *M* → ∞, the model copes well with sparse inputs.

### Behavioral-level learning using the SPP learning in a closed-loop setup

To further investigate the network effects of the SPP learning rule, we implemented a closed loop setup where a recurrent spiking neural network controls a behaving agent. So far, we have treated the pre-synaptic firing times that trigger the synaptic PSC release as externally given, resulting in the reduced model where synapses only control post-synaptic firing. By considering a recurrent network, the synapse also indirectly gains control over pre-synaptic firing times (see Section 9 in S1 Appendix). The behavioral setup is illustrated in Fig 5A. A fixed goal position *x*_*goal*_ has to be reached starting from *x*_*start*_ in a 3-dimensional task space. The network that was used to learn this task is shown in Fig 5B. A set of input neurons encoded representations of the current position of the agent’s end effector, which are connected to a recurrent network of internal state neurons. A set of action neurons were selected from the recurrent network to encode movement directions that are applied to update the agent’s position. The total network firing rate was controlled via feedback inhibition. All excitatory synapses in the network were trained using the SPP learning rule Eq (11). During training, actions are given externally to provide a supervisor signal. The training trajectory had a duration of 1 s. 8 example movement trajectories after learning for 220 seconds are shown in Fig 5B. Fig 5C shows corresponding network activity for a single trajectory after learning. The network has learned internal representations to reliably control the agent’s end effector in a closed loop setting.

Next, we evaluated the effect of variability in synaptic transmission on learning performance. To test this, we repeated the above experiment with different release probability parameters *r*_0_ and evaluated the learning performance. The performance was evaluated based on the mean squared error (MSE) between the target trajectory (see dark blue curve Fig 5B for reference) and the trajectories generated by the network after learning. The results are shown in Fig 5D. Interestingly, the best performance was not achieved with deterministic synapses (*r*_0_ = 1), but noisy synapses consistently showed lower MSE. This result shows that stochastic synaptic release in the SPP learning framework, introduced here, can be beneficial also at the behavioral level.

## Discussion

Predictive processing and the related theory of free energy minimization has been praised for its ability to describe biological phenomena on different spatial and temporal scales [16, 40]. Here we started from the lower end of the spatial hierarchy: individual synapses that learn to interact with their postsynaptic neuron. To the best of our knowledge, this is the first time that PP is applied to the subcellular level to derive local learning rules, proposing a new role of stochastic pre-synaptic release. Previous models suggest that pre-synaptic noise increases the energy and information transmission efficiency of synapses [12, 41–44]. We show that stochastic pre-synaptic release can also be utilized to encode uncertainty about the somatic membrane potential, providing a theory on the role of noise on the postsynaptic side. We also demonstrate a first step investigating the functional implication of SPP on the network and behavioral level, by demonstrating that stochastic synaptic release is beneficial for solving the closed-loop reaching task in Fig 5. A possible explanation for this phenomenon is that noisy release in SPP increases the robustness of the behavioral loop, by including more variability in the training data.

These findings complement previous results to derive detailed properties of neural networks based on PP [45, 46]. In our SPP framework, in order to predict the membrane potential from limited information, synapses must encode their uncertainty about the true state of the membrane potential. Our hypothesis is that uncertainty is expressed as variability and manifests itself as trial-by-trial noise in synaptic transmission. Furthermore, we show that at the macroscopic level, synapses learn to encode probability distributions over neuronal firing times, which can be retrieved after learning.

### Prior related work

Predictive processing has been very successful in explaining animal behavior and brain function [16, 47–49]. On the neuron and network level, previous models utilized PP to derive learning rules for reward-based learning and models of the dopaminergic system [50]. [20] used PP to derive synaptic weight updates with third factor modulation using dopamine-like signals. A number of previous studies have approached the problem of deriving learning rules from related variational Bayesian inference theory [51–55] and information-theoretic measures [56–59]. In [21] a model for dendritic prediction of somatic spiking was proposed. Different to the SPP approach, the uncertainty about the somatic membrane potential was not represented in these models.

Recently, it was shown that PP may also provide an interesting alternative to the error back-propagation algorithm for learning in deep neural networks [60, 61]. The SPP complements these results with a bottom-up approach for spiking networks. An important property of the SPP learning rule is that synaptic updates only depend on the timing of pre- and post-synaptic spikes, which makes the model well suited for event-based neural simulation [62, 63] and new brain-inspired hardware [64, 65]. Therefore, SPP learning is a promising candidate to control unreliable signal transmission in diverse neuromorphic technologies [7–9] and even to exploit the unreliability for learning.

### Testable experimental predictions

Direct experimental evidence for predictive coding in cultured neurons was provided by [66]. In [67] a PP-based encoding model was formulated and could account for observed electrophysiological responses in vitro. Evidence for predictive coding is also abundantly available in *in vivo* recordings of neural activity and brain anatomy [68–72]. However, PP has often been criticized for being too general to make any falsifiable experimental predictions [73]. In contrast, the SPP proposed here makes very precise predictions about the interplay of synaptic and neural dynamics. Our model directly predicts that synapses should be stochastic to effectively encode uncertainties about the somatic membrane potential. Furthermore, the SPP makes precise predictions about the synaptic plasticity changes in post-pre-post spike pairing protocols and the dependence on the synaptic weight (Fig 2).

Throughout our derivations and experiments, we have used the LIF neuron model, with delta pulse synaptic currents, for simplicity. However, the approach presented here is in principle compatible with any spiking neuron model with complex membrane and synapse dynamics. However, if more complex models are considered, the stochastic bridge model used to describe the membrane potential and synaptic current dynamics between two spike times, will most likely no longer have a closed-form solution. In these cases, a numerical approximation of the stochastic bridge could be used. Furthermore, the convenient triplet rule property is lost if the state of the neuron is not perfectly reset at spike times, as in the simple LIF model considered here. The study of these more complex cases will be the subject of future work.

## Conclusion

In summary, we have presented a synapse-centric account of PP that views synapses as agents that interact with their post-synaptic neuron much like an organism interacts with its environment. The emerging SPP learning rule is qualitatively consistent with experimentally observed synaptic mechanisms while being analytically tractable. Our results complement previous applications of PP on the system and network level, and demonstrates that manifestations of PP can be identified even on the smallest scales of brain function. In contrast to this prior work, our model synapses use only local information and yields triplet STDP dynamics which can be directly tested against experiments. The emergent learning algorithm is fully event-based, i.e., computation only takes place when pre- and post-synaptic spikes arrive at the synapses. The model is therefore very well suited for event-based neural simulation and brain-inspired hardware.

## Supporting information

S1 Appendix Details of derivation and implementation of the SPP model. First, we review the main idea behind predictive processing (PP) and how it is utilized here on the level of single synapses and related mathematical derivations. Then, we describe details about our main theoretical result showing that the synaptic efficacy updates minimize the free energy of the synaptic efficacy with respect to the back-propagating action potentials. Furthermore, we show that the same learning rules also emerge if the SPP is applied to a learning scenario for recurrent neural networks with arbitrary numbers of neurons and synapses.
(PDF)
