# Supplementary material for: Synapses learn to utilize stochastic pre-synaptic release for the prediction of postsynaptic dynamics
Source: PLoS Comput Biol. 2024 Nov 4;20(11):e1012531. doi: 10.1371/journal.pcbi.1012531 (PMC11534197; doi:10.1371/journal.pcbi.1012531)
Supplement: S1 Appendix — First, we review the main idea behind predictive processing (PP) and how it is utilized here on the level of single synapses and related mathematical derivations. Then, we describe details about our main theoretical result showing that the synaptic efficacy updates minimize the free energy of the synaptic efficacy with respect to the back-propagating action potentials. Furthermore, we show that the same learning rules also emerge if the SPP is applied to a learning scenario for recurrent neural networks with arbitrary numbers of neurons and synapses. (PDF) [file pcbi.1012531.s001.pdf]

S1 Appendix of the manuscript "Synapses learn to utilize stochastic pre-synaptic release for the prediction of postsynaptic dynamics"

David Kappel[1,2] and Christian Tetzlaff[1,3]

In this Supplemental Material, we provide the details to the derivation and implementation of the SPP model. This document is organized as follows: In Section 1 we review the main idea behind predictive processing (PP) and how it is utilized here on the level of single synapses. In Section 2 we define the *generative density* that is used by the synapse to estimate the state of the somatic membrane potential. In Section 3 we define the *recognition density* that determines the dynamics of the stochastic synapse model. In Sections 4-7 we establish useful properties of the model. In Section 8 we develop our main theoretical result to show that the synaptic efficacy updates (Eq. 10 in main text), minimize the free energy $\mathcal{F}(z,w)$ of the synaptic efficacy w with respect to the back-propagating action potentials z. In Section 9 we show that the same learning rules also emerge if the SPP is applied to a learning scenario for recurrent neural networks with arbitrary numbers of neurons and synapses.

1 Synapse-level predictive processing

Here we provide a brief overview over the main aspects of PP and free energy minimization that are needed for our treatment of single synapses. Since we focus here on a relatively simple physical system – individual synapses that interacts with their post-synaptic neuron – we only need a subset of the theoretical framework that is provided by PP. An excellent comprehensive review on this topic can be found in [1].

PP is a generic theoretical framework to describe the interaction of a behaving agent with its environment. Its main assumption is that the agent and the environment have physically separated states, the *internal* (w) and *external* (u) states, respectively, that cannot directly influence another. Interaction only takes place through specific *actions* (y), performed by the agent and *feedback* (z) provided by the environment. PP suggests that the agent should adapt its behavior to minimize the surprise caused by the feedback z, measured by the negative log likelihood, $\mathrm{surprise}(z) = -\log p(z)$. PP proposes a specific method to approaching a state of minimum surprise. This method rests on the idea that a biological organism maintains an internal model of its environment, that allows it to infer statistical properties of the external states u. The internal model is composed of two parts, (1) the *recognition density* $q(u \mid w)$, that describes how the external state u interacts with the internal state w, and (2) the *generative density* $p(u,z)$, that describes the dependency between external states u and feedback z [1]. To simplify the

notation we employ here the commonly used shortcut $q_w(u)$ for $q(u \mid w)$. The recognition density is parameterized by the internal state w, and the generative density depends in our model on the set of somatic parameters that comprise the firing threshold ϑ, reset- and resting potential, u_r and u_0, and the membrane time constant τ_m.

Using this internal model, the complexity of the surprise minimization problem can be approached by replacing the goal to minimize surprise directly by a variational upper bound, that allows us to split the problem into two parts. The theory stems from the observation that an upper bound on the surprise can be reached indirectly by employing the recognition density q to *guess* external states u, and the generative density p evaluates how well the feedback z agrees with the guessed external states u. The problem to minimize surprise is then augmented with a divergence term to also minimizing the mismatch between q and p.

We adopted this idea and suggest minimizing an upper bound on the surprise in every synapse, given by the variational free energy $\mathcal{F}$, which is defined as

$$\mathcal{F}(z,w) \;=\; \text{surprise}(z) + \text{divergence}(q|p) \;=\; -\log p(z) \;+\; \mathcal{D}_{\text{KL}}\big(q \,\|\, p\big) \quad\geq\quad \text{surprise}(z)\,, \tag{1}$$

where $\mathcal{D}_{\text{KL}}\big(q \,\|\, p\big)$ is the Kullback-Leibler (KL)-divergence between $q_w(u)$ and $p(u \mid z)$. The inequality in (1) follows from $\mathcal{D}_{\text{KL}}\big(q \,\|\, p\big) \geq 0$ for any two probability distributions q and p. Inserting the definition of the KL-divergence, we get

$$\mathcal{F}(z,w) \;=\; -\log p(z) + \mathcal{D}_{\text{KL}}\big(q \,\|\, p\big) \tag{2}$$

$$\;=\; -\log p(z) + \left\langle \log \frac{q_w(u)}{p(u \mid z)} \right\rangle_{q_w(u)} \;=\; \left\langle \log \frac{q_w(u)}{p(u, z)} \right\rangle_{q_w(u)}\,, \tag{3}$$

where $\left\langle f(u) \right\rangle_{q_w(u)}$ denotes the expectation of some function $f(u)$ with respect to the probability density $q_w(u)$. By rearranging the terms of this last form, we can establish a link to the Helmholtz free energy that measures the useful energy potential in closed thermodynamic systems [1,2], by interpreting $\varepsilon(u,z) = -\log p(u,z)$ as the energy of state (u,z), to get

$$\underbrace{\mathcal{F}(z,w)}_{\text{Variational free energy}} \;=\; \underbrace{\left\langle \varepsilon(u,z) \right\rangle_{q_w(u)}}_{\text{Expected internal energy with respect to } q_w} \;-\; \underbrace{H(q_w)}_{\text{Entropy of } q_w}\,. \tag{4}$$

Using these definitions, we identify the relevant variables in our synapse model that are required by PP: (1.) *the internal states*, (2.) *the actions*, (3.) *the external states*, and (4.) the *feedback* (see [3] and Fig. 1 in main text for an illustration).

1. *The internal states* summarizes all relevant internal variables that determine the behavior of the synapse. Since we focus here on long-term synaptic plasticity, the internal state is given by the synaptic efficacy w. The internal states can be augmented with additional variables to also include other mechanisms, e.g. short term plasticity, but we neglect these here for the sake of simplicity.

Although we peruse here a treatment of PP that focuses on single synapses, we will find it useful to consider the vector of all synapses projecting to one post-synaptic neuron, which we write as $\mathbf{w}$. We will use corresponding variants of the recognition density $q_{\mathbf{w}}(u)$ and the free energy $\mathcal{F}(z, \mathbf{w})$ where necessary.

2. *The actions* are utilized by synapses to interact with the environment (the efferent neuron). In our model, this is done through stochastic synaptic currents y, where the mean and variance of y is governed by the synaptic efficacy w. In our model, y denotes a sequence of synaptic currents $y = (y(t) \mid t \geq 0)$.

3. *The external states.* From the perspective of a synapse, the environment, it can immediately interact with, is the post-synaptic neuron. Here, we model the external states as the somatic membrane potential $u(t)$ of a leaky integrate and fire (LIF) neuron with firing threshold ϑ and resting potential u_0. We denote the whole sequence of the somatic membrane potential by $u = (u(t) \mid t \geq 0)$.

4. *The feedback.* In our model, a synapse only receives the back-propagating action potential of the post-synaptic neuron z as feedback to be informed about the somatic membrane potential. Formally, the spike train z is denoted by the set of firing times $t_n^{\text{post}}, t_{n+1}^{\text{post}}, \ldots$ of the post-synaptic neuron. This feedback information about the external state $u(t)$ is used by the synapse to update the internal model of the environment $p(u, z)$.

Learning is realized here by minimizing $\mathcal{F}(z, w)$ with respect to w, which can be done by gradient descent

$$\Delta w \; = \; -\frac{\partial}{\partial w}\mathcal{F}(z, w) \; , \tag{5}$$

In the following sections we will derive the learning rule that solves this optimization problem for the case of our synapse model step by step. We consider the general form of the weight changes Δw to show that the learning problem (5) can be solved by applying weight updates that only depend on the pre- and post-synaptic firing times, the current value of the synaptic efficacy and constants that are independent of learning, given by

$$\Delta w \; = \; W_3(\Delta t_1, \Delta t_2, w) \; = \; W_{\text{LTP}}(\Delta t_1, \Delta t_2) \; - \; \left(\frac{1 - r_0}{2\, r_0} + w\right) W_{\text{LTD}}(\Delta t_1, \Delta t_2) \; + \; \frac{1}{2\, w} \, , \tag{6}$$

with $\Delta t_1 = t_2^{\text{post}} - t^{pre}$ and $\Delta t_2 = t_2^{\text{post}} - t_1^{\text{post}}$. (6) is the general case for an arbitrary synaptic parameter r_0. Eq. 10 in the main text shows the special case for $r_0 = \frac{1}{2}$ which was used throughout the paper (except Fig. 4E). In our simulations we performed synaptic efficacy updates $w_{new} = w_{old} + \eta \, \Delta w$ for every post-pre-post spike triplet, with $t_1^{\text{post}} < t^{pre} < t_2^{\text{post}}$, where t_1^{post} and t_2^{post} are the spike times of two neighboring post-synaptic spikes, and t^{pre} is a pre-synaptic spike time. η is a small positive constant

learning rate $\eta = 10^{-5}$. Weight updates were applied immediately at the arrival of the bAP events t_2^{post} (no batching or buffering).

Throughout this paper we assume that the somatic parameters, that determine the generative density, are constant and encoded *a-priori* into the dynamics of the synapses such that the dynamics of the soma and the synapse match (e.g. through evolutionary processes or adaptation that is significantly slower than the learning dynamics). Plasticity mechanisms to fine-tune the synaptic behavior to track changes in somatic parameters could be derived from the SPP framework as well, but are not the focus of this study.

2 The generative density

In this section we formally define the generative density $p(u, z)$ which describes the behavior of the environment, i.e. the joint dynamics of the membrane potential u, and the observed post-synaptic spike train z back-propagating to the synapse, in Eq 3. To do so, we consider the accumulated input currents that arrive at a given neuron over the time interval of length T in the general form

$$\mathcal{Y}_M(t) = \sum_{m=1}^{\lfloor tM \rfloor} y_m \,, \tag{7}$$

where M is here a parameter that scales the number of inputs that arrive in the interval T and y_m denote individual input events of finite amplitude. $\mathcal{Y}_M(t)$ may contain numerous noisy inputs, including inhibitory and excitatory synaptic PSC inputs, as well as other noise sources such as stochastic opening and closing of ion channels.

When considering a physical system with a large number of noisy inputs, such as a neuron, it is convenient to study the limit $M \to \infty$. For example, by Donsker's theorem, if the input currents $y_m = \frac{1}{\sqrt{M}}\epsilon_m$ are given by independent random variables ϵ_m with mean 0 and variance 1, which is the limit of infinitely many and infinitely small pre-synaptic inputs, $\mathcal{Y}_M(t)$ approaches the Wiener process $\mathcal{W}(t)$, i.e. $\lim_{M\to\infty} \mathcal{Y}_M(t) = \mathcal{W}(t)$. This limit is the basis of many stochastic variants of spiking neuron models and reflects the fact that neurons in the brain have typically an abundance of noisy inputs, where each individual input only has a small effect on the dynamics of the membrane potential (e.g. see Chapter 2.2. of [4], Chapter 7 of [5] and earlier models [6–8]). Some of these related models make a detour via the Poisson process and use the Kurtz approximation [4, 9] to arrive at the Wiener process $\mathcal{W}(t)$ to model neuronal inputs.

Here we also adopt this model, which allows us to rewrite the dynamics of the membrane potential $u(t)$ (Eq. 1 in main text) in terms of a stochastic differential equation, by setting the input current $y(t) = d\mathcal{W}(t)$, to get

$$du = \frac{1}{\tau_m}(u_0 - u(t))\, dt + \sigma_0\, d\mathcal{W}(t) \,, \tag{8}$$

with resting membrane potential u_0 and where σ_0 scales the contribution of the total stochastic input

and $d\mathcal{W}(t)$ are the increments of the Wiener process.

Eq. (8) is an Ornstein-Uhlenbeck (OU)-process that describes the dynamics of the LIF neuron model with stochastic inputs [5]. This model is convenient because it compactly captures the uncertainty about $u(t)$ and all inputs to the post-synaptic neuron. Furthermore, the OU process can be solved analytically using stochastic calculus, e.g. if the process (8) is fixed to u_0 at time 0 it evolves according to

$$u(t) \;=\; u_0 \;+\; \sigma_0 \int_0^t e^{-\frac{t-s}{\tau_m}} d\mathcal{W}(s)\,. \tag{9}$$

For long observation times the OU process converges to a stationary distribution, given by a Gaussian with mean u_0 and constant variance.

Furthermore, Eq. (8) can be used to model the joint distribution $p\,(u,z)$ by solving the first passage time problem to retrieve a distribution over post-synaptic firing times z alongside u, which has been extensively studied (e.g. [7, 10, 11]). This model, albeit being relatively simple, has proven to predict the spiking behavior of biological neurons quite well [12–14]

3 The recognition density

Here, we define the recognition density $q_{\mathbf{w}}\,(u)$ for our synapse model. The recognition density describes the interaction between synaptic efficacies $\mathbf{w}$ and the membrane potential u through PSCs . Synapses inject brief current pulses into the postsynaptic neuron when a pre-synaptic input arrives. Let the spike times of pre-synaptic neurons in a given time window T be denoted by t_m^{pre}. The post-synaptic input current $y(t)$ is defined for $0 \le t \le T$ as

$$y(t) \;=\; \sum_{m=1}^{M} \delta\,(t_m^{\text{pre}} - t)\; y_m\,, \quad \text{with} \quad y_m = \tfrac{1}{M}\omega_m \quad \text{and} \quad \omega_m = r_0\, w_{i(m)} + \sqrt{s_0\, w_{i(m)}}\,\epsilon_m\;, \tag{10}$$

where ω_m are the PSC amplitudes, $i(m)$ maps the PSC index m to the index of the source synapse that generated the current event ϵ_m and where $w_i > 0$ is the synaptic efficacy. This is equivalent to Eq. 8 in main text, but it makes the stochastic contribution explicit. The constants r_0 and s_0 scale, the mean and variance of the synaptic current. M denotes here the total number of inputs that arrive in the interval T. As before, we have $\epsilon_m \sim \mathcal{N}\,(\epsilon_m\,|\,0,1)$. If arrival rates of pre-synaptic inputs are assumed to be constant in T we have $\mathcal{Y}_M(t) = \int_0^t y(s)ds$, from Eq. (7)[1]. We are interested in the case $r_0 > 0$ and $s_0 > 0$, where y_m's have a deterministic and a stochastic contribution. We used $r_0 = \frac{1}{2}$ and $s_0 = r_0\,(1 - r_0)$ if not stated otherwise in accordance with previous models [15]. Our Gaussian model, (10), is an approximation to this previous model of stochastic synaptic release.

[1] We interpret the Dirac delta function $\delta(t)$ as the distributional derivative of the Heaviside step function here and in the remainder of the treatment.

We can use the above results to define the recognition density $q_{\mathbf{w}}(\mathbf{y})$

$$y_m \sim q_{\mathbf{w}}(y_m) = \mathcal{N}\left(y_m \,\middle|\, \tfrac{1}{M} r_0\, w_{i(m)}, \tfrac{1}{M^2} s_0\, w_{i(m)}\right) \quad \text{and} \quad q_{\mathbf{w}}(\mathbf{y}) = \prod_m q_{\mathbf{w}}(y_m) \,. \tag{11}$$

To determine the recognition density $q_{\mathbf{w}}(u)$ we use that the somatic membrane dynamics can be described through a deterministic function g, with $u(t) = g(y,t)$, that maps a given sequence of synaptic input currents, y, to the current value of the membrane potential at time t. g is a piece-wise continuous function that obeys the membrane dynamics (Eqs. 1-3 in main text). The recognition density for synaptic currents $q_{\mathbf{w}}(\mathbf{y})$ can thus be used to generate the somatic membrane potential using

$$u(t) = g(y,t) = u_0 + \frac{R}{\tau_m} \int_0^t e^{-\frac{t-s}{\tau_m}} y(s)\, ds \,, \tag{12}$$

with y given by (10) and $\mathbf{y} \sim q_{\mathbf{w}}(\mathbf{y})$.

4 Stochastic bridge model of the membrane potential dynamics

As we have seen in Section 2, the OU process (8) dynamics can be used to define the generative density $p(u,z)$ for a LIF neuron that receives a large number of noisy inputs. In the derivation of the SPP we make use of the fact, that also the posterior density $p(u \mid z)$ can be solved explicitly. The information about the spike times z deflects the distribution of likely values of the membrane potential from its resting state, which is expressed in the posterior density $p(u \mid z)$. We can express this posterior as a so-called stochastic bridge model [16], that determines the dynamics of $u(t)$ given the information that the membrane potential is at the firing threshold ϑ at the firing times t^{post}, i.e., the constraint $u(t_1^{\text{post}}) = u_r$ and $u(t_2^{\text{post}}) = \vartheta$ through a stochastic process with time varying mean $\mu(t)$ and variance $\sigma^2(t)$. Hence, we use a Gaussian process model of the external state, such that the posterior density is given by

$$p(u(t) \mid z) = \mathcal{N}\left(u(t) \,\middle|\, \mu(t), \sigma^2(t)\right) \,. \tag{13}$$

Using the SPP theory we can in principle assume any function $\mu(t)$ and $\sigma^2(t)$ and develop learning rules that will strive to best approximate its dynamics. However, a reasonable choice will obey the constraints imposed by the neuron and synapse dynamics, e.g., the membrane time constant and the firing mechanism and resetting behavior of the neuron.

For LIF neuron model (8) the resulting *constraint stochastic process* has to fulfill the following requirements

1. The mean $\mu(t)$ obeys $\mu(t_1^{\text{post}}) = u_r$ and $\mu(t_2^{\text{post}}) = \vartheta$.
2. For $t_1^{\text{post}} < t < t_2^{\text{post}}$, $\mu(t)$ approaches the resting potential u_0 asymptotically.
3. The variance $\sigma^2(t)$ obeys $\sigma^2(t) \geq -\frac{\tau_m}{2}\left(\sigma^2(t)\right)'$ for $t_1^{\text{post}} < t < t_2^{\text{post}}$, and approaches its minimum when close to the firing times t_1^{post} and t_2^{post}.

4. For $t_1^{\text{post}} < t \leq t_2^{\text{post}}$, $\sigma^2(t)$ approaches the variance σ_0^2 of the stationary distribution asymptotically.
5. The functions $\mu(t)$ and $\sigma^2(t)$ are smooth and follow the LIF dynamics with time constant τ_m.

Constraint 3. incorporates that in the LIF dynamics, the variance can only shrink at a maximum speed proportional to the membrane time constant τ_m. $\left(\sigma^2(t)\right)' = \frac{d}{dt}\sigma^2(t)$ denotes the time derivative.

The LIF neuron implies OU process dynamics of the membrane potential. Given the information that the membrane potential is at the firing threshold ϑ at the firing times t^{post}, i.e., the constraint $u(t_1^{\text{post}}) = u_r$ and $u(t_2^{\text{post}}) = \vartheta$, the OU process can be solved explicitly. The solution to this double constraint stochastic process is the OU-bridge process [16, 17]. For any neighboring postsynaptic spike pair $(t_1^{\text{post}}, t_2^{\text{post}})$ and time point t with, $t_1^{\text{post}} < t \leq t_2^{\text{post}}$ we can describe the dynamics of $u(t)$ using its mean $\mu(t)$ and variance $\sigma^2(t)$. Using this result, for any neighboring postsynaptic spike pair $(t_1^{\text{post}}, t_2^{\text{post}})$ and time point t with $t_1^{\text{post}} < t \leq t_2^{\text{post}}$ we describe the dynamics of $u(t)$ using the mean $\mu(t)$ and variance function $\sigma^2(t)$, given by

$$\mu(t) = \left\langle u(t) \right\rangle = \mu\left(\Delta t_1, \Delta t_2\right) = u_0 + (u_r - u_0)\frac{e^{\frac{\Delta t_1}{\tau_m}} - e^{-\frac{\Delta t_1}{\tau_m}}}{e^{\frac{\Delta t_2}{\tau_m}} - e^{-\frac{\Delta t_2}{\tau_m}}} + (\vartheta - u_0)\frac{e^{\frac{\Delta t_2 - \Delta t_1}{\tau_m}} - e^{\frac{\Delta t_1 - \Delta t_2}{\tau_m}}}{e^{\frac{\Delta t_2}{\tau_m}} - e^{-\frac{\Delta t_2}{\tau_m}}} \tag{14}$$

and

$$\sigma^2(t) = \left\langle u^2(t) \right\rangle - \left\langle u(t) \right\rangle = \sigma^2\left(\Delta t_1, \Delta t_2\right) = \sigma_0^2 \frac{1}{1 + \gamma\left(e^{\frac{\Delta t_1 - \Delta t_2}{\tau_m}} + e^{-\frac{\Delta t_1}{\tau_m}}\right)}, \tag{15}$$

where $\Delta t_1 = t_2^{\text{post}} - t$, $\Delta t_2 = t_2^{\text{post}} - t_1^{\text{post}}$ and γ is a constant that scales the slope of the variance function. In other words, the dynamics of the membrane potential subject to the constraint $u(t_1^{\text{post}}) = u_r$ and $u(t_2^{\text{post}}) = \vartheta$ are described by a stochastic process with mean $\mu(t)$ and variance $\sigma^2(t)$. The membrane potential mean and variance functions (14) and (15) are piece-wise defined for all postsynaptic spike intervals $(t_n^{post}, t_{n+1}^{post})$. The membrane dynamics during each interval are statistically independent of each other due to the resetting behavior of the neuron model. In all simulations, we used $\gamma = 50$ and $\sigma_0^2 = 16$.

The mean function $\mu(t)$ in (14) is identical to the OU-bridge process model [16, 17]. This function describes the asymptotic approach to the resting potential u_0 and the slope towards the firing threshold ϑ. The variance function $\sigma^2(t)$ in (15) is flatter than the direct solution of the OU-bridge process model to incorporate the additional constraint 3.

Using the definition (14) and (15), we find that the posterior of the generative density can be evaluated at any time point t, as $p\left(u(t) \mid z\right) = \mathcal{N}\left(u(t) \,\middle|\, \mu(t), \sigma^2(t)\right)$. Furthermore, since the mean (14) and variance (15) functions only depend on the relative spike timing Δt_1 and Δt_2 we find that these quantities can be expressed in the form $\mu\left(\Delta t_1, \Delta t_2\right)$ and $\sigma^2\left(\Delta t_1, \Delta t_2\right)$.

5 PSC posterior distribution

At this point it is instructive to note that the generative density, that was introduced in Section 2, can be solved directly for the PSCs y_m. More precisely, we will show that $p\left(y \mid z\right)$ can be expressed as a Gaussian distribution with time-varying mean and variance functions $m(t)$ and $v(t)$, such that $y_m \sim \mathcal{N}\left(y_m \,\middle|\, \frac{1}{M}m(t_m), \frac{1}{M^2}v(t_m)\right)$. Therefore, the generative density can be inverted, yielding a time varying distribution over PSCs $y(t)$ that, when injected into the post-synaptic neuron, will give the desired distribution for the membrane potential dynamics. This result can be obtained by stochastic integration, but to keep this paper self-contained we provide a proof here. We start by considering a general drift-diffusion process and then show the special case of the LIF neuron dynamics step by step. This suggests that the posterior distribution $p\left(u \mid z\right)$ can be constructed by drawing PSCs from a suitable distribution $y_m \sim p\left(y_m \mid t_m^{\text{pre}}, z\right)$ and then using (12) to generate u.

In general, the evolution of the probability density function $p(u,t)$ of a stochastic process u at time t, with drift $A(u,t)$ and diffusion $B(u,t)$, can be described by the Fokker-Planck equation

$$\frac{\partial}{\partial t}p(u,t) = -\frac{\partial}{\partial u}\left(A(u,t)\cdot p(u,t)\right) + \frac{1}{2}\frac{\partial^2}{\partial u^2}\left(B(u,t)\cdot p(u,t)\right) . \tag{16}$$

Note that u denotes here an instantaneous value rather than whole sequences. To describe the dynamics of our model neuron we treat the case where $p(u,t)$ is a Gaussian distribution with time-varying mean $\mu(t)$ and variance $\sigma^2(t)$ functions, i.e. $u(t) \sim \mathcal{N}\left(u(t) \,\middle|\, \mu(t), \sigma^2(t)\right)$ at any time point t, to get for the left-hand side of (16)

$$\frac{\partial}{\partial t}p(u,t) = p(u,t)\left(\mu'(t)\frac{u-\mu(t)}{\sigma^2(t)} + \frac{1}{2}\left(\sigma^2(t)\right)'\left(\frac{(u-\mu(t))^2}{\sigma^4(t)} - \frac{1}{\sigma^2(t)}\right)\right) ,$$

where $\mu'(t) = \frac{d}{dt}\mu(t)$ and $\left(\sigma^2(t)\right)' = \frac{d}{dt}\sigma^2(t)$ denote the time derivatives. Furthermore, we can expand the right-hand side of (16) to get

$$\frac{\partial}{\partial u}\left(A(u,t)\cdot p(u,t)\right) = p(u,t)\left(\frac{\partial}{\partial u}A(u,t) - A(u,t)\frac{u-\mu(t)}{\sigma^2(t)}\right)$$

and

$$\frac{\partial^2}{\partial u^2}\left(B(u,t)\cdot p(u,t)\right) = \\ p(u,t)\left(\frac{\partial^2}{\partial u^2}B(u,t) - 2\frac{\partial}{\partial u}B(u,t)\frac{u-\mu(t)}{\sigma^2(t)} + B(u,t)\left(\frac{(u-\mu(t))^2}{\sigma^4(t)} - \frac{1}{\sigma^2(t)}\right)\right) .$$

Therefore, by plugging these results back into the Fokker-Planck equation (16), we find the condition

that has to be satisfied for functions $A(u,t)$ and $B(u,t)$ to be given by

$$\begin{aligned} \mu'(t)\frac{u-\mu(t)}{\sigma^2(t)} + \frac{1}{2}\left(\sigma^2(t)\right)'\left(\frac{(u-\mu(t))^2}{\sigma^4(t)} - \frac{1}{\sigma^2(t)}\right) &\overset{!}{=} \\ A(u,t)\frac{u-\mu(t)}{\sigma^2(t)} - \frac{\partial}{\partial u}A(u,t) + \frac{1}{2}\frac{\partial^2}{\partial u^2}B(u,t) &- \\ \frac{\partial}{\partial u}B(u,t)\frac{u-\mu(t)}{\sigma^2(t)} + \frac{1}{2}B(u,t)\left(\frac{(u-\mu(t))^2}{\sigma^4(t)} - \frac{1}{\sigma^2(t)}\right) &. \end{aligned} \tag{17}$$

6 Solution for the LIF neuron model

This last results (17) holds in general. To arrive at the final result, we replace the general drift-diffusion dynamics with the special case of a current-based leaky integrate and fire neuron model with finite integration time constant τ_m using the ansatz $A(u,t) = \frac{1}{\tau_m}(u_0 - u) + m(t)$ and $B(u,t) = v(t)$. In this case, we can make condition (17) satisfied if $\mu'(t) = \frac{1}{\tau_m}(u_0 - \mu(t)) + m(t)$ and $\left(\sigma^2(t)\right)' = v(t) - \frac{2}{\tau_m}\sigma^2(t)$. This can be verified by plugging this result back into (17)

$$\begin{aligned} \left(\frac{1}{\tau_m}(u_0-\mu(t)) + m(t)\right)\frac{u-\mu(t)}{\sigma^2(t)} + \frac{1}{2}\left(v(t) - \frac{2}{\tau_m}\sigma^2(t)\right)\left(\frac{(u-\mu(t))^2}{\sigma^4(t)} - \frac{1}{\sigma^2(t)}\right) &\overset{!}{=} \\ \left(\frac{1}{\tau_m}(u_0-u) + m(t)\right)\frac{u-\mu(t)}{\sigma^2(t)} + \frac{1}{\tau_m} + \frac{1}{2}v(t)\left(\frac{(u-\mu(t))^2}{\sigma^4(t)} - \frac{1}{\sigma^2(t)}\right) &, \end{aligned}$$

from which the equality follows

$$\begin{aligned} \leftrightarrow\quad & \frac{1}{\tau_m}(u_0-\mu(t))\frac{u-\mu(t)}{\sigma^2(t)} - \frac{1}{\tau_m}\left(\frac{(u-\mu(t))^2}{\sigma^2(t)} - 1\right) \overset{!}{=} \frac{1}{\tau_m}(u_0-u)\frac{u-\mu(t)}{\sigma^2(t)} + \frac{1}{\tau_m} \\ \leftrightarrow\quad & (u_0-\mu(t))\frac{u-\mu(t)}{\sigma^2(t)} - \frac{(u-\mu(t))^2}{\sigma^2(t)} \overset{!}{=} (u_0-u)\frac{u-\mu(t)}{\sigma^2(t)} \\ \leftrightarrow\quad & (u_0-u)\frac{u-\mu(t)}{\sigma^2(t)} \overset{!}{=} (u_0-u)\frac{u-\mu(t)}{\sigma^2(t)} \qquad \square \end{aligned}$$

This proofs that a stochastic process u with $p(u,t) = \mathcal{N}\left(u(t)\,\middle|\,\mu(t),\sigma^2(t)\right)$, $\mu'(t) = \frac{1}{\tau_m}(u_0 - \mu(t)) + m(t)$ and $\left(\sigma^2(t)\right)' = v(t) - \frac{2}{\tau_m}\sigma^2(t)$ is realized by a drift $A(u,t) = \frac{1}{\tau_m}(u_0 - u) + m(t)$ and diffusion $B(u,t) = v(t)$. Equivalently, any process u with mean $\mu(t)$ and variance $\sigma^2(t)$ can be realized if

$$\begin{aligned} m(t) &= \mu'(t) + \frac{1}{\tau_m}(\mu(t) - u_0)\ , \\ v(t) &= \left(\sigma^2(t)\right)' + \frac{2}{\tau_m}\sigma^2(t)\ , \end{aligned} \tag{18}$$

and $v(t) \geq 0$ can be satisfied for all t. This last result is used in Section 8 to derive the learning rule (6).

Furthermore, by integration of this last result we find that any integrable functions $m(t)$ and $v(t) > 0$ yield the following dynamics for the stochastic process u

$$\begin{aligned} \mu(t) &= u_0 + e^{-\frac{t}{\tau_m}} \int_0^t e^{\frac{s}{\tau_m}} m(s)\, ds \\ \sigma^2(t) &= e^{-\frac{2t}{\tau_m}} \int_0^t e^{\frac{2s}{\tau_m}} v(s)\, ds \,. \end{aligned} \tag{19}$$

For $m(t) = 0$ and $v(t) = v_0$ (constant) we recover the Ornstein-Uhlenbeck process dynamics.

Therefore, we define the posterior density $p(y \mid z)$ as the distribution over y where any instantaneous value y_m at time t_m obeys

$$p(y(t) \mid z) = \mathcal{N}(y(t) \mid m(t), v(t)) \,, \tag{20}$$

A sampling-based approximation for $y(t)$ is provided by sampling y_m with mean $\frac{1}{M} m(t_m)$ and variance $\frac{1}{M} v(t_m)$ according to (18). We can then use (10) to recover instantaneous values for $y(t)$. This approximation becomes exact for $M \to \infty$. Finally we can recover samples from $p(u \mid z)$ using samples from $y_m \sim p(y_m \mid t_m, z)$ and then using $u(t) = g(y, t)$.

Our model of synaptic currents (10) and (11) suggests that the standard deviation is scaled by $\frac{1}{M}$, thus using the defintion of the recognition density we find a suitable formulation for the posterior for PSC events

$$p(y_m \mid t_m, z) = \mathcal{N}\left(y_m \,\middle|\, \tfrac{1}{M} m(t_m), \tfrac{1}{M^2} v(t_m)\right) \,, \tag{21}$$

where $m(t)$ and $v(t)$ are as defined in (18) with $\mu(t)$ and $\sigma^2(t)$ given by the solution to $p(u \mid z)$ as defined in (14) and (15), respectively. This formulation has the advantage that the variance of the effective posterior is re-scaled by $\frac{1}{M}$ and thus very reliable firing behavior can be realized (in the limit $M \to \infty$ even deterministic). The parameter σ_0^2 can be used to scale the neural variability relative to any finite value of M.

7 Spurious agency of individual synapses

Another useful property of the formulation (7) is its resilience to perturbations, i.e., the influence of any finite PSC event y_m vanishes compared to the large number of noisy inputs. This becomes clear by taking the derivative of (12) with respect to the synaptic efficacy w_i of a single SPC event y_m. By considering the chain rule for derivatives, we get

$$\frac{\partial u}{\partial w_i} = \frac{\partial u}{\partial \mathcal{Y}_M} \frac{\partial \mathcal{Y}_M}{\partial \omega_m} \frac{\partial \omega_m}{\partial w_i} = \frac{\partial u}{\partial \mathcal{Y}_M} \frac{1}{M} \frac{\partial \omega_m}{\partial w_i} \,, \quad \text{and thus} \quad \lim_{M \to \infty} \frac{\partial u}{\partial w_i} = 0 \,. \tag{22}$$

In practice also any finite sum of PSC events will have negligible effect on the membrane dynamics, suggesting that a single synapse has only a small effect on the neuron dynamics that vanishes for large

M. This *spurious agency* of a single synapse may appear as a nuisance, but it is a very useful property that formalizes the fact that a single synapse has minuscule control over the behavior of the post-synaptic neuron. It requires the entirety of the combined pre-synaptic input $\mathcal{Y}_M(t)$ to significantly manipulate the post-synaptic firing behavior. One of the main technical contributions of our analysis is to demonstrate how spurious agency can be exploited to derive simple, local learning rules to learn the parameters of a stochastic process $\mathcal{Y}_M(t)$ such that a desired firing behavior is installed in the post-synaptic neuron.

8 Derivation of the learning rule

Next we make use of the result from Sections 2 and 3 to develop the learning rules that minimize the variational free energy (3). The approach that is taken here makes extensive use of the fact that the generative density $p(u, z)$ can be expressed analytically, and Bayesian posteriors can be solved in closed form. Using the results and definitions outlined above, (5) becomes

$$\Delta w = -\frac{\partial}{\partial w}\mathcal{F}(z, \mathbf{w}) = \frac{\partial}{\partial w}\left\langle \log\frac{p(u, z)}{q_{\mathbf{w}}(u)}\right\rangle_{q_{\mathbf{w}}(u)} \tag{23}$$

$$= \frac{\partial}{\partial w}\left\langle \log\frac{p(u \mid z)}{q_{\mathbf{w}}(u)}\right\rangle_{q_{\mathbf{w}}(u)} + \frac{\partial}{\partial w}\log\left\langle p(u, z)\right\rangle_u \tag{24}$$

The equality follows from Bayes rule. The last term is constant in u and can therefore be pulled out of the expectation. Furthermore, we find that it becomes zero for $M \to \infty$ using the spurious agency property established in Section 7. To see this, we expand the term to get

$$\frac{\partial}{\partial w}\log\left\langle p(u, z)\right\rangle_u = \frac{\left\langle \frac{\partial}{\partial w}p(u, z)\right\rangle_u}{\left\langle p(u', z)\right\rangle_{u'}} = \left\langle \frac{\partial}{\partial w}p(u \mid z)\right\rangle_u = \frac{1}{M}\left\langle \frac{\partial}{\partial u}p(u \mid z)\,\frac{\partial u}{\partial y_m}\frac{\partial \omega_m}{\partial w}\right\rangle_u, \tag{25}$$

where we used $\frac{\partial y_m}{\partial \omega_m} = \frac{1}{M}$. The gradient Eq 25 shrinks to zero as M approaches large values. Thus, we can write

$$\Delta w = -\frac{\partial}{\partial w}\mathcal{F}(z, \mathbf{w}) \approx \frac{\partial}{\partial w}\left\langle \log\frac{p(u \mid z)}{q_{\mathbf{w}}(u)}\right\rangle_{q_{\mathbf{w}}(u)} = -\frac{\partial}{\partial w}\mathcal{D}_{\mathrm{KL}}\big(q_{\mathbf{w}}(u)\,\|\,p(u \mid z)\big)\,. \tag{26}$$

$\mathcal{D}_{\mathrm{KL}}\big(q\,\|\,p\big)$ is the Kullback-Leibler (KL)-divergence between the posterior according to the generative density and the recognition density. The approximation can be made arbitrarily tight by using large numbers of synapses per neurons, which corresponds to large M in practice. In the limit, $M \to \infty$ the approximation becomes exact. M was chosen large but finite in our simulations to achieve a good approximation ($M = 1000\,\mathit{spikes/s}$ if not stated otherwise).

Finally, we exploit that the KL-divergence is invariant to parameter transformations (see e.g. Theorem 1 in [18]), using the invertible mapping $u(t) = g(y, t)$, abbreviated $u = g(y)$ for the whole sequence,

we get

$$\Delta w = -\frac{\partial}{\partial w}\mathcal{F}(z,\mathbf{w}) = \frac{\partial}{\partial w}\left\langle \log\frac{p(u\mid z)}{q_{\mathbf{w}}(u)}\right\rangle_{q_{\mathbf{w}}(u)} = \frac{\partial}{\partial w}\left\langle \log\frac{p(g(y)\mid z)}{q_{\mathbf{w}}(g(y))}\right\rangle_{q_{\mathbf{w}}(g(y))}$$
$$= \frac{\partial}{\partial w}\left\langle \log\frac{p(y\mid z)}{q_{\mathbf{w}}(y)}\right\rangle_{q_{\mathbf{w}}(y)} = -\frac{\partial}{\partial w}\mathcal{D}_{\mathrm{KL}}\big(q_{\mathbf{w}}(y)\,\|\,p(y\mid z)\big)\,. \tag{27}$$

Thus, the minimization of the variational free energy (1) with respect to the synaptic efficacy w of a synapse that interacts with its post-synaptic neuron, can be reduced to a gradient on the mismatch between the posterior synaptic current y given z and the recognition density $q_{\mathbf{w}}(y)$. Finally, we use that the PSC pulses in y are independent and thus the marginal in (27) can be replaced by a sum over pre-synaptic firing times t_m^{pre}. This allows us to factorize Eq. (27) to get

$$\Delta w = -\frac{\partial}{\partial w}\mathcal{F}(z,\mathbf{w}) = \sum_m \frac{\partial}{\partial w}\left\langle \log\frac{p(y_m\mid t_m^{\mathrm{pre}},z)}{q_{\mathbf{w}}(y_m)}\right\rangle_{q_{\mathbf{w}}(y_m)}$$
$$= -\sum_m \frac{\partial}{\partial w}\mathcal{D}_{\mathrm{KL}}\big(q_{\mathbf{w}}(y_m)\,\|\,p(y_m\mid t_m^{\mathrm{pre}},z)\big)\,. \tag{28}$$

Also note, that it is sufficient here to consider only the two post-synaptic spikes in z, that are directly neighboring t_m^{pre}, to evaluate $p(y_m\mid t_m^{\mathrm{pre}},z)$, since the somatic membrane dynamics are independent after the reset to u_r.

This result is useful, because the generative model established in Section 2 allows us to express the posterior distribution $p(y_m\mid t_m,z)$ in closed form. In Section 3 we show in detail that Gaussian synaptic currents $y(t)$ with mean $m(t_m)$ and variance $v(t_m)$ enables a large set of synapses to realize a somatic membrane potential $u(t)$ that obeys the stochastic processes with mean $\mu(t)$ and variance $\sigma^2(t)$, if

$$\begin{aligned} m(t) &= \mu'(t) + \frac{1}{\tau}\left(\mu(t) - u_0\right)\ , \\ v(t) &= \left(\sigma^2(t)\right)' + \frac{2}{\tau}\sigma^2(t)\ , \end{aligned} \tag{29}$$

where $\mu(t)$ and $\sigma^2(t)$ are as defined in (14) and (15).

To construct the term $\frac{\partial}{\partial w}\left\langle \log\frac{p(y_m\mid t_m,z)}{q_{\mathbf{w}}(y_m)}\right\rangle_{q_{\mathbf{w}}(y)}$ of (28) we use the result from Section 2 and assume

a general Gaussian form $y_m \sim \mathcal{N}\left(y_m \,\middle|\, \mu_w, \sigma_w^2\right)$ to get

$$\begin{aligned}
&\frac{\partial}{\partial w}\left\langle \log\frac{p\left(y_m \mid t_m, z\right)}{q_{\mathbf{w}}\left(y_m\right)} \right\rangle_{q_{\mathbf{w}}(y_m)} \\
&= \frac{\partial}{\partial w}\left\langle -\frac{1}{2}\log\left(\frac{2}{M^2}\pi\, v(t_m)\right) - \frac{\left(y_m - \frac{1}{M}m(t_m)\right)^2}{\frac{2}{M^2}v(t_m)} \right\rangle_{q_{\mathbf{w}}(y_m)} + \frac{1}{2}\frac{\partial}{\partial w}\log\left(2\,\pi\, e\,\sigma_w^2\right) \\
&= \frac{\partial}{\partial w}\left\langle \frac{\frac{2}{M}\,y_m\, m(t_m) \; - \; y_m^2}{\frac{2}{M^2}\,v(t_m)} \right\rangle_{q_{\mathbf{w}}(y_m)} + \frac{1}{2}\frac{1}{\sigma_w^2}\frac{\partial}{\partial w}\sigma_w^2 \\
&= \left(M\,\frac{m(t_m)}{v(t_m)}\right)\frac{\partial}{\partial w}\left\langle y_m \right\rangle_{q_{\mathbf{w}}(y_m)} - \frac{1}{2}\left(\frac{M^2}{v(t_m)}\right)\frac{\partial}{\partial w}\left\langle y_m^2 \right\rangle_{q_{\mathbf{w}}(y_m)} + \frac{1}{2}\frac{1}{\sigma_w^2}\frac{\partial}{\partial w}\sigma_w^2 \,. \qquad (30)
\end{aligned}$$

By plugging in (14) and (15) we recover the LTP and LTD term in (6).

Using $\left\langle y_m \right\rangle_{q_{\mathbf{w}}(y_m)} = \mu_w$ and $\left\langle y_m^2 \right\rangle_{q_{\mathbf{w}}(y_m)} = \mu_w^2 + \sigma_w^2$, we get

$$\frac{\partial}{\partial w}\left\langle \log\frac{p\left(y_m \mid t_m, z\right)}{q_{\mathbf{w}}\left(y_m\right)} \right\rangle_{q_{\mathbf{w}}(y_m)} = \left(\frac{M\, m(t_m) - M^2\,\mu_w}{v(t_m)}\right)\frac{\partial}{\partial w}\mu_w - \frac{1}{2}\left(\frac{M^2}{v(t_m)} - \frac{1}{\sigma_w^2}\right)\frac{\partial}{\partial w}\sigma_w^2 \,. \qquad (31)$$

Finally, using (10) we identify μ_w and σ_w^2 to get the result for any t_m at the pre-synaptic firing times

$$\begin{aligned}
\frac{\partial}{\partial w}\left\langle \log\frac{p\left(y_m \mid t_m, z\right)}{q_{\mathbf{w}}\left(y_m\right)} \right\rangle_{q_{\mathbf{w}}(y_m)} &= \left(M\,\frac{m(t_m) - r_0\, w}{v(t_m)}\right)\frac{r_0}{M} - \frac{1}{2}\left(\frac{M^2}{v(t_m)} - \frac{M^2}{w\, r_0\,(1-r_0)}\right)\frac{r_0}{M^2}\,(1-r_0) \\
&= r_0\,\frac{m(t_m)}{v(t_m)} - r_0^2\,\frac{1}{v(t_m)}\left(\frac{1-r_0}{2\,r_0} + w\right) + \frac{1}{2\,w}\,, \qquad (32)
\end{aligned}$$

which is identical to the result in (6) with $W_{\text{LTP}}(\Delta t_1, \Delta t_2) = r_0\,\frac{m(t_m)}{v(t_m)}$ and $W_{\text{LTD}}(\Delta t_1, \Delta t_2) = r_0^2\,\frac{1}{v(t_m)}$. Note, that this result is independent of M and thus does not vanish (or explode) in the limit to large numbers of synapses.

Using this we identify the triplet STDP windows, given by

$$W_{\text{LTP}}(\Delta t_1, \Delta t_2) = r_0\,\frac{\mu'\left(\Delta t_1, \Delta t_2\right) + \frac{1}{\tau}\left(\mu\left(\Delta t_1, \Delta t_2\right) - u_0\right)}{\left(\sigma^2\left(\Delta t_1, \Delta t_2\right)\right)' + \frac{2}{\tau}\sigma^2\left(\Delta t_1, \Delta t_2\right)}\,, \qquad (33)$$

and

$$W_{\text{LTD}}(\Delta t_1, \Delta t_2) = r_0^2\,\frac{1}{\left(\sigma^2\left(\Delta t_1, \Delta t_2\right)\right)' + \frac{2}{\tau}\sigma^2\left(\Delta t_1, \Delta t_2\right)}\,, \qquad (34)$$

where $\mu\left(\Delta t_1, \Delta t_2\right)$ and $\sigma^2\left(\Delta t_1, \Delta t_2\right)$, respectively, are the estimated mean and variance of the membrane potential based on the back-propagating action potentials ((14) and (15)), and $\mu'(t) = \frac{d}{dt}\mu(t)$ and $\left(\sigma^2(t)\right)' = \frac{d}{dt}\sigma^2(t)$ denote the time derivatives.

The rational underlying the learning model is illustrated in Fig. 2A. For any pre-synaptic firing time,

t_m a random PSC is generated using the recognition density $q_{\mathbf{w}}(\epsilon_m)$. In addition, post-pre-post spike triplets are formed by considering the neighboring post-synaptic spikes back-propagating to the synapse. Based on these spike triplets, the posterior density $p(\epsilon_m \mid t_m, z)$ is constructed and the mismatch with the recognition density triggers a weight update. The internal model does not need to be represented explicitly but is implicit in the shape of the triplet STDP learning window.

The proposed SPP learning rules installs a single parameter distribution $q_{\mathbf{w}}(y)$ that minimizes the distance to the two-parameter posterior density $y \sim \mathcal{N}(y \mid m(t), v(t))$. In Fig. 3E we showed that the synaptic efficacies are correlated with μ^* and σ^*. Using the result (32) we can make a more careful analysis, by keeping the PSC posterior fixed, $m(t) = \mu^*$ and $v(t) = (\sigma^*)^2$, and then analyzing the roots of the learning rule Eq. (32). Using this we find that the weights converge to $w^* = \frac{1}{2}\left(\mu^* - \frac{1}{2}\right) + \frac{1}{2}\sqrt{\left(\mu^* - \frac{1}{2}\right)^2 + (2\sigma^*)^2}$, (with $r_0 = \frac{1}{2}$ as in our simulations). Hence, weights encode both the target mean and variances, i.e., for small σ^* and large μ^* we have $w^* \approx \mu^*$ and for large σ^* and small μ^* we have $w^* \approx \sigma^*$. From this fixed-point solution, it also becomes clear that $w^* \geq 0$ holds in general, which follows directly from $\sqrt{\left(\mu^* - \frac{1}{2}\right)^2 + (2\sigma^*)^2} \geq |(\mu^* - \frac{1}{2})| \geq 0$.

9 Network level SPP

In this section, we turn to a network-level treatment of the SPP, to show that the same learning rules (26) can be also applied to a class of learning problems and networks of neurons with an arbitrary number of neurons and synapses. To show this we use here vectorized forms $\mathbf{z} = (z_1, \ldots, z_K)$, $\mathbf{u} = (u_1, \ldots, u_K)$, $\mathbf{y} = (y_1, \ldots, y_K)$ and $\mathbf{w} = (w_{11}, \ldots, w_{KK})$ to denote ordered sets of network spikes, membrane potentials, PSCs and the synaptic efficacies, respectively, for networks of K neurons. w_{ki} denotes the synaptic efficacy from neuron i to neuron k.

Furthermore, we assume that there is an additional external feedback $\mathbf{x}$ that may influence the activity of an arbitrary subset of network neurons. This feedback is assumed to be a sensory experience of some form that is directly perceptible by a subset of the neurons. The feedback may take the role of a teacher signal as in our example in Fig. 5, but it can also be a more abstract signal that indicates, e.g., goal reaching or aversive stimuli. Subsequently, we will take the prediction error minimization problem to the network level by considering the network spikes $\mathbf{z}$ as part of the state space and minimizing the augmented variational free energy

$$\mathcal{F}(\mathbf{x}, \mathbf{w}) = \left\langle \log \frac{q_{\mathbf{w}}(\mathbf{u}, \mathbf{z})}{p(\mathbf{u}, \mathbf{x}, \mathbf{z})} \right\rangle_{q_{\mathbf{w}}(\mathbf{u}, \mathbf{z})} . \tag{35}$$

The network-level generative density. The corresponding generative density $p(\mathbf{u}, \mathbf{x}, \mathbf{z})$ now captures the dependence between states $(\mathbf{u}, \mathbf{z})$ and feedback $\mathbf{x}$. $\mathbf{x}$ can in principle be an arbitrary vector-valued function of time, but as in the single neuron case we focus here on models where the posterior density can be solved and results in conditional independence between individual membrane potentials

u_k

$$p(\mathbf{u} \mid \mathbf{x}, \mathbf{z}) = \prod_k p(u_k \mid \mathbf{x}, \mathbf{z}) \ . \tag{36}$$

The following derivations will be valid for scenarios where this posterior density can be evaluated. $\mathbf{x}$ can for example be given by strong external inputs that impose a certain firing pattern on a subset of network neurons or a bias potential that offsets the resting potential of some neurons. Scenarios where the independence (36) does not hold lead to more complex learning rules (see the discussion at the end of this section).

As in the single synapse case, we can make use of another conditional independence that is imposed by the neuron's resetting behavior at post-synaptic firing times. This implies conditional independence between any two post-synaptic spike times $t^{\text{post}}_{k,n-1}, t^{\text{post}}_{k,n}$. To make this explicit, we denote by $u_{k,n} = u_k(t)$ and $\mathbf{z}_{k,n} = \mathbf{z}(t)$, $t \in [t^{\text{post}}_{k,n-1}, t^{\text{post}}_{k,n})$, i.e. the subsets of the sequences u_k and $\mathbf{z}$, respectively, over the time interval $t^{\text{post}}_{k,n-1} \leq t < t^{\text{post}}_{k,n}$. Using this, we can write for (36)

$$p(\mathbf{u} \mid \mathbf{x}, \mathbf{z}) = \prod_{k,n} p\left(u_{k,n} \mid \mathbf{x}, \mathbf{z}_{k,n}\right) \ . \tag{37}$$

The network-level recognition density. For the single synapse neuron, we assumed the pre-synaptic spikes to be given, which considerably simplified the derivations. This is not possible anymore for the network-level model, which also has to reflect the recurrent input from network spikes $\mathbf{z}$ from other neurons. To account for this additional dynamics, we consider the network-level PSCs, $y_k(t)$ of neuron k

$$y_k(t) = \sum_{i \setminus k} y_{ki}(t) = \sum_{i \setminus k, m} \delta\left(t^{\text{pre}}_{i,m} - t\right) y_{ki,m} \ , \quad \text{with} \quad q_{w_{ki}}\left(y_{ki,m}\right) = \mathcal{N}\left(y_{ki,m} \mid r_0\, w_{ki}, s_0\, w_{ki}\right) \ , \tag{38}$$

$$\text{and} \qquad q_{\mathbf{w}}\left(y_{k,n} \mid \mathbf{z}_{k,n}\right) = \prod_{i \setminus k} \prod_{m:\, t^{\text{post}}_{k,n-1} \leq t^{\text{pre}}_{i,m} < t^{\text{post}}_{k,n}} q_{w_{ki}}\left(y_{ki,m}\right) \tag{39}$$

where the sum over i runs over all network neurons excluding k (fully recurrent network without autapses). $y_{ki,m}$ denotes the PSC amplitude of the m-th PSC event under synapse ki, and $y_{k,n}$ denotes the whole PSC sequence of neuron k in the time interval $t^{\text{post}}_{k,n-1} \leq t^{\text{pre}}_{i,m} < t^{\text{post}}_{k,n}$. The pre-synaptic firing times $t^{\text{pre}}_{i,m} = t^{\text{post}}_{i,m} + T_s$, denote the recurrent network activity with some small time delay T_s.

The network-level recognition density determines the response of the network to the PSCs (39). To define the network-level recognition density, we can make use of the fact that the neuron model is Markovian, which allows us to define $q_{\mathbf{w}}(\mathbf{u}, \mathbf{z})$ in terms of a distribution over individual network spike

times $t^{\text{post}}_{k,n}$

$$q_{\mathbf{w}}\left(\mathbf{u},\mathbf{z}\right) = \prod_{k,n} q_{\mathbf{w}}\left(u_{k,n},\, t^{\text{post}}_{k,n} \,\middle|\, \mathbf{z}_{k,n}\right) = \prod_{k,n} \left\langle p\left(u_{k,n} \,\middle|\, y'_{k,n}\right) p\left(t^{\text{post}}_{k,n} \,\middle|\, y'_{k,n}\right) q_{\mathbf{w}}\left(y'_{k,n} \,\middle|\, \mathbf{z}_{k,n}\right) \right\rangle_{y'_{k,n}} ,$$

$$\text{and equivalently} \quad q_{\mathbf{w}}\left(\mathbf{y},\mathbf{z}\right) = \prod_{k,n} p\left(t^{\text{post}}_{k,n} \,\middle|\, y_{k,n}\right) q_{\mathbf{w}}\left(y_{k,n} \,\middle|\, \mathbf{z}_{k,n}\right) , \tag{40}$$

where $p\left(t^{\text{post}}_{k,n} \,\middle|\, y_{k,n}\right)$ is the probability density over firing time $t^{\text{post}}_{k,n}$ in response to given PSCs $y_{k,n}$, given by a point density, where the membrane potential reaches the firing threshold exactly at time $t^{\text{post}}_{k,n}$.

Using this result we get for the network level synaptic efficacy updates

$$-\frac{\partial}{\partial w_{li}} \mathcal{F}\left(\mathbf{x},\mathbf{w}\right) = \frac{\partial}{\partial w_{li}} \left\langle \log \frac{p\left(\mathbf{u},\mathbf{x},\mathbf{z}\right)}{q_{\mathbf{w}}\left(\mathbf{u},\mathbf{z}\right)} \right\rangle_{q_{\mathbf{w}}(\mathbf{u},\mathbf{z})} \approx \frac{\partial}{\partial w_{li}} \left\langle \log \frac{p\left(\mathbf{u},\mathbf{z} \,|\, \mathbf{x}\right)}{q_{\mathbf{w}}\left(\mathbf{u},\mathbf{z}\right)} \right\rangle_{q_{\mathbf{w}}(\mathbf{u},\mathbf{z})} \tag{41}$$

$$= \frac{\partial}{\partial w_{li}} \left\langle \sum_{k,n} \log \frac{p\left(u_{k,n},\, t^{\text{post}}_{k,n} \,\middle|\, \mathbf{x},\mathbf{z}_{k,n}\right)}{q_{\mathbf{w}}\left(u_{k,n},\, t^{\text{post}}_{k,n} \,\middle|\, \mathbf{z}_{k,n}\right)} \right\rangle_{q_{\mathbf{w}}(\mathbf{u},\mathbf{z})} = \Delta w_{li} , \tag{42}$$

where the approximation follows the same reasoning as in (26) and becomes tight for large numbers of pre-synaptic spikes. Again, by exploiting the properties of (40) we can use reparameterization to replace the marginal over u with one over y. For (42) we get

$$\Delta w_{li} = \frac{\partial}{\partial w_{li}} \left\langle \sum_{k,n} \log \frac{p\left(u_{k,n},\, t^{\text{post}}_{k,n} \,\middle|\, \mathbf{x},\mathbf{z}_{k,n}\right)}{q_{\mathbf{w}}\left(u_{k,n},\, t^{\text{post}}_{k,n} \,\middle|\, \mathbf{z}_{k,n}\right)} \right\rangle_{q_{\mathbf{w}}(\mathbf{u},\mathbf{z})} =$$

$$\frac{\partial}{\partial w_{li}} \left\langle \sum_{k,n} \log \frac{\left\langle p\left(g\left(y_{k,n}\right) \,\middle|\, y'_{k,n}\right) p\left(t^{\text{post}}_{k,n} \,\middle|\, y'_{k,n}\right) p\left(y'_{k,n} \,\middle|\, \mathbf{x},\mathbf{z}_{k,n}\right) \right\rangle_{y'_{k,n}}}{\left\langle p\left(g\left(y_{k,n}\right) \,\middle|\, y'_{k,n}\right) p\left(t^{\text{post}}_{k,n} \,\middle|\, y'_{k,n}\right) q_{\mathbf{w}}\left(y'_{k,n} \,\middle|\, \mathbf{z}_{k,n}\right) \right\rangle_{y'_{k,n}}} \right\rangle_{q_{\mathbf{w}}(\mathbf{y},\mathbf{z})} =$$

$$\frac{\partial}{\partial w_{li}} \left\langle \sum_{k,n} \log \frac{p\left(y_{k,n} \,|\, \mathbf{x},\mathbf{z}_{k,n}\right)}{q_{\mathbf{w}}\left(y_{k,n} \,|\, \mathbf{z}_{k,n}\right)} \right\rangle_{q_{\mathbf{w}}(\mathbf{y},\mathbf{z})} . \tag{43}$$

Note that the information about $\mathbf{z}$ is redundantly encoded in $\mathbf{u}$ and therefore this dependence vanishes under the expectation. Finally, using this result and (39), we identify the synaptic efficacy updates for w_{li}

$$\Delta w_{li} = \frac{\partial}{\partial w_{li}} \left\langle \sum_{k,n} \log \frac{p\left(y_{k,n} \,|\, \mathbf{x},\mathbf{z}_{k,n}\right)}{q_{\mathbf{w}}\left(y_{k,n} \,|\, \mathbf{z}_{n}\right)} \right\rangle_{q_{\mathbf{w}}(\mathbf{y},\mathbf{z})} =$$

$$\frac{\partial}{\partial w_{li}} \left\langle \sum_{n} \log \frac{p\left(y_{l,n} \,|\, \mathbf{x},\mathbf{z}_{l,n}\right)}{q_{\mathbf{w}}\left(y_{l,n} \,|\, \mathbf{z}_{n}\right)} \right\rangle_{q_{\mathbf{w}}(\mathbf{y},\mathbf{z})} =$$

$$\frac{\partial}{\partial w_{li}} \left\langle \sum_{n} \sum_{m:\, t^{\text{post}}_{l,n-1} < t^{\text{pre}}_{i,m} \leq t^{\text{post}}_{l,n}} \log \frac{p\left(y_{li,m} \,\middle|\, t^{\text{pre}}_{i,m},\, \mathbf{x},\, \mathbf{z}_{l,n}\right)}{q_{w_{li}}\left(y_{li,m}\right)} \right\rangle_{q_{\mathbf{w}}(\mathbf{y},\mathbf{z})} , \tag{44}$$

where $p\left(y_{li,m} \,\middle|\, t_{i,m}^{\text{pre}}, \mathbf{x}, \mathbf{z}_{l,n}\right)$ is the posterior density over PSCs evaluated at time $t_{i,m}^{\text{pre}}$.

A sampling-based approximation of (44) can be realized by, first, sampling $\mathbf{y}, \mathbf{z} \sim q_{\mathbf{w}}(\mathbf{y}, \mathbf{z})$ by letting the network evolve according to its intrinsic dynamics. Then, update the synaptic efficacies for every post-pre-post spike according to

$$\Delta w_{li} = \frac{\partial}{\partial w_{li}} \left\langle \log \frac{p\left(y_{li,m} \,\middle|\, t_{i,m}^{\text{pre}}, \mathbf{x}, \mathbf{z}_{l,n}\right)}{q_{w_{li}}\left(y_{li,m}\right)} \right\rangle_{q_{w_{li}}(y_{li,m})} . \quad (45)$$

In summary, we find that network-level learning can be established in the form (45) if the posterior of the generative density $p\left(y_{li,m} \,\middle|\, t_{i,m}^{\text{pre}}, \mathbf{x}, \mathbf{z}_{l,n}\right)$ can be computed in the synapses (realized through the learning rules). This assumption is clearly only true for special cases where the implied conditional independence holds. In Fig. 4 and 5 we chose the most basic example for this to be true. There, we used a simple model where $\mathbf{x}$ provided external input to drive a subset of neurons to fire at externally defined time points (e.g., through external sensory inputs that provide strong inputs to some neurons). In this simple example $p\left(y_{li,m} \,\middle|\, t_{i,m}^{\text{pre}}, \mathbf{x}, \mathbf{z}_{l,n}\right)$ in (45) can be expressed analogously to Eq. (20) and the resulting learning rules are identical to Eq. 10 in main text with the only difference that spike times are generated by the intrinsic dynamics $q_{\mathbf{w}}(\mathbf{y}, \mathbf{z})$ for neurons that are not driven externally by $\mathbf{x}$. In more complex scenarios the posterior can be realized through an additional learning mechanism, e.g., through neuromodulatory signals, as previously studied in [19].

References

1. Buckley CL, Kim CS, McGregor S, Seth AK. The free energy principle for action and perception: A mathematical review. Journal of Mathematical Psychology. 2017;81:55-79.

2. Neal R, Hinton G. A view of the EM algorithm that justifies incremental sparse, and other variants. Learning in Graphical Models. 1998:355-68.

3. Friston K. Variational filtering. NeuroImage. 2008;41(3):747-66.

4. Greenwood PE, Ward LM. Stochastic neuron models. vol. 1. Springer; 2016.

5. Gerstner W, Kistler WM, Naud R, Paninski L. Neuronal dynamics: From single neurons to networks and models of cognition. Cambridge University Press; 2014.

6. Gerstein GL, Mandelbrot B. Random walk models for the spike activity of a single neuron. Biophysical journal. 1964;4(1):41-68.

7. Lansky P, Smith CE, Ricciardi L. One-dimensional stochastic diffusion models of neuronal activity and elated first passage time problems. North Carolina State University. Dept. of Statistics; 1989.

8. Sacerdote L, Giraudo MT. Stochastic integrate and fire models: a review on mathematical methods and their applications. Stochastic biomathematical models. 2013:99-148.

9. Kurtz TG. Strong approximation theorems for density dependent Markov chains. Stochastic Processes and their Applications. 1978;6(3):223-40.

10. Ricciardi LM, Sacerdote L. The Ornstein-Uhlenbeck process as a model for neuronal activity. Biological cybernetics. 1979;35(1):1-9.

11. Ricciardi LM, Sato S. First-passage-time density and moments of the Ornstein-Uhlenbeck process. Journal of Applied Probability. 1988;25(1):43-57.

12. Jolivet R, Rauch A, Lüscher HR, Gerstner W. Predicting spike timing of neocortical pyramidal neurons by simple threshold models. Journal of computational neuroscience. 2006;21(1):35-49.

13. Clopath C, Jolivet R, Rauch A, Lüscher HR, Gerstner W. Predicting neuronal activity with simple models of the threshold type: Adaptive exponential integrate-and-fire model with two compartments. Neurocomputing. 2007;70(10-12):1668-73.

14. Jolivet R, Kobayashi R, Rauch A, Naud R, Shinomoto S, Gerstner W. A benchmark test for a quantitative assessment of simple neuron models. Journal of neuroscience methods. 2008;169(2):417-24.

15. Katz B. Quantal mechanism of neural transmitter release. Science. 1971;173(3992):123-6.

16. Corlay S. Properties of the Ornstein-Uhlenbeck bridge; 2013. ArXiv preprint arXiv:1310.5617.

17. Szavits-Nossan J, Evans MR. Inequivalence of nonequilibrium path ensembles: the example of stochastic bridges. Journal of Statistical Mechanics: Theory and Experiment. 2015;2015(12):P12008.

18. Qiao Y, Minematsu N. A study on invariance of f-divergence and its application to speech recognition. IEEE Transactions on Signal Processing. 2010;58(7):3884-90.

19. Isomura T, Sakai K, Kotani K, Jimbo Y. Linking neuromodulated spike-timing dependent plasticity with the free-energy principle. Neural computation. 2016;28(9):1859-88.
